# Assembly of ceria-Nrf2 nanoparticles as macrophage-targeting ROS scavengers protects against myocardial infarction

**DOI:** 10.3389/fphar.2024.1503757

**Published:** 2025-01-10

**Authors:** Wenjing Liao, Jinduan Lin, Wenli Wang, Ming Zhang, Yanfang Chen, Xin Li, Huan Liu, Pan Xia Wang, Guojun Zhao, Jijun Fu, Xiaoqian Wu

**Affiliations:** ^1^ The Sixth Affiliated Hospital, Guangzhou Municipal and Guangdong Provincial Key Laboratory of Molecular Target and Clinical Pharmacology, the NMPA and State Key Laboratory of Respiratory Disease, School of Pharmaceutical Sciences, Guangzhou Medical University, The Fifth Affiliated Hospital, Guangzhou, China; ^2^ Department of Pharmacy, Guangzhou Eighth People’s Hospital, Guangzhou Medical University, Guangzhou, China; ^3^ State Key Laboratory of Experimental Hematology, Institute of Hematology and Blood Diseases Hospital, Chinese Academy of Medical Sciences and Peking Union Medical College, Tianjin, China

**Keywords:** myocardial infarction, ceria nanoparticles, Nrf2, oxidative stress, inflammation

## Abstract

Myocardial infarction (MI) is a leading cause of morbidity and mortality worldwide, and mitigating oxidative stress is crucial in managing MI. Nuclear factor erythroid 2-related factor 2 (Nrf2) plays a critical role in combating oxidative stress and facilitating cardiac remodeling post-MI. Here, we engineered Cerium oxide (CeO_2_) nanoparticle-guided assemblies of ceria/Nrf2 nanocomposites to deliver Nrf2 plasmids. The CeO_2_/Nrf2 nanocomposites effectively activated the Nrf2/antioxidant response element (ARE) signaling pathway both *in vivo* and *in vitro*. In a mouse MI model induced by permanent ligation of the left anterior descending artery (LAD), CeO_2_/Nrf2 nanocomposites were administered via tail vein injection, predominantly targeting circulating monocytes and macrophages which will be recruited to the heart post MI due to the acute inflammatory response. We demonstrated that CeO_2_/Nrf2 nanocomposites alleviated cardiac systolic dysfunction and significantly reduced infarct size and scar fibrosis post-MI. Furthermore, CeO_2_/Nrf2 nanocomposites effectively mitigated MI-induced oxidative stress and downregulated Nrf2-regulated inflammatory genes (tumor necrosis factor-α, IL-6, and inducible nitric oxide synthase), thereby reducing cardiomyocyte apoptosis. These findings indicate that CeO_2_/Nrf2 nanocomposites significantly enhance Nrf2 signaling activation and confer protection against MI. This study identifies CeO_2_/Nrf2 nanocomposites as a promising strategy for post-MI therapy.

## Graphical Abstract

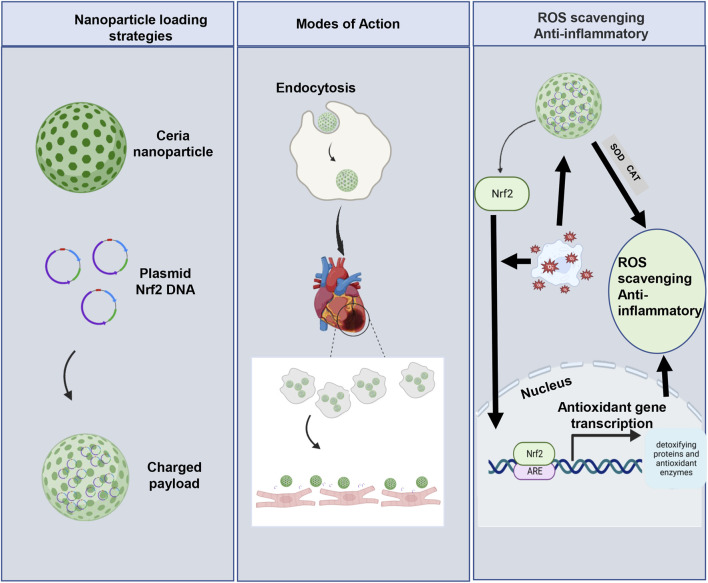

## 1 Introduction

Acute myocardial infarction (AMI) remains a leading cause of mortality and morbidity worldwide, accounting for approximately 40%–50% of sudden cardiac deaths (Rallidis et al., 2022). Although prompt restoration of blood flow is a potential strategy, reperfusion increases the incidence of cardiac dysfunction and cardiomyocyte death (Méndez-Valdés et al., 2022; Dalkara and Arsava, 2012). Cardiomyocytes, being terminally differentiated cells, are particularly susceptible to acute or persistent ischemia, leading to irreversible injury, pathological cardiac remodeling, and sudden death (Liu et al., 2021). Despite decades of research, discovering ideal therapeutic strategies for cardiac protection and repair following ischemia remains challenging.

Numerous studies have revealed that oxidative stress and inflammatory responses are key contributors to myocardial injury and pathological remodeling (Chouchani et al., 2014; Forman and Zhang, 2021). Elevated levels of reactive oxygen species (ROS) induce oxidative stress, promoting myocardial cell death and cardiac dysfunction. As a crucial regulator of redox balance, nuclear factor erythroid 2-related factor 2 (Nrf2) governs the transcription of downstream antioxidant enzymes (Huang et al., 2022; Shen et al., 2019). Under physiological conditions, Nrf2 is retained in the cytoplasm but translocates to the nucleus in response to oxidative stress (Shen et al., 2019; Pi et al., 2008). By activating the transcription of over 200 genes, including antioxidant enzymes, Nrf2 plays a pivotal role in defending against oxidative stress. Previously, we demonstrated that the natural product Urolithin B protects against myocardial ischemia by enhancing nuclear translocation of Nrf2 in response to oxidative stress and inflammatory stimuli (Zheng et al., 2020). Activation of Nrf2 can mitigate irreversible cardiomyocyte damage by regulating intracellular redox signaling pathways (Huang et al., 2022; Bubb et al., 2017). We and others demonstrated that Nrf2 is a potential target for the clinical treatment and prevention of myocardial infarction injury (Mills et al., 2018; Wu et al., 2022; Gao et al., 2022). However, an efficient Nrf2 activator remains unavailable, particularly for cardiovascular disease treatment. Therefore, effective delivery of Nrf2 to the heart is critical for improving myocardial infarction outcomes.

Nanoparticles have emerged as promising materials with diverse functionalities (El Khoury et al., 2021; Sun and Hou, 2023), and cerium oxide nanoparticles, or nanoceria, exhibit significant antioxidant potential (Saifi et al., 2021; Lord et al., 2021). Nanoceria is gaining recognition as a potent antioxidant in various pathological contexts. However, its application in cardiovascular disease remains challenging, particularly in targeted drug delivery. Following myocardial infarction (MI), dying cardiomyocytes trigger a robust inflammatory response characterized by the infiltration of inflammatory cells, particularly monocytes/macrophages (Lavine et al., 2018; Seropian et al., 2014). Macrophages, a type of innate immune cell, infiltrate the infarcted area, phagocytize dead cells, and promote angiogenesis and scar formation. Macrophages produce both pro-inflammatory (TNF-α, IL-1β, and proteases) and anti-inflammatory (IL-10, TGF-β) cytokines. Molecular tomography has shown the recruitment of monocytes and macrophages to the infarcted myocardium (Heo et al., 2019). Notably, within 48 h post-AMI, macrophages are mobilized from the bone marrow and recruited to the infarcted area (Chen et al., 2023). This property makes macrophages an attractive vehicle for noninvasive imaging and targeted drug delivery to the infarcted myocardium.

One challenge of using nucleic acids as therapeutic agents is their rapid degradation as well as their highly negative charge, which can reduce cellular uptake (Kumar et al., 2023; Sharma et al., 2022). Nanotechnology offers innovative methods to stabilize nucleic acid molecules (Tu et al., 2023). Nanoparticles have emerged as a desirable strategy with diverse functionalities, and cerium oxide nanoparticles, or nanoceria, are a premium choice due to their antioxidant potential (Mohamed, 2022; Casals et al., 2020). Cerium oxide (CeO_2_) nanoparticles, with low toxicity and good biocompatibility, have been investigated as artificial enzymes and drug delivery materials (Xu et al., 2013; Li et al., 2013). In a recent study, Wang et al. demonstrated that CeO_2_ nanoparticles protect against cardiomyocyte apoptosis and ischemia/reperfusion (I/R) injury in mice (Wang et al., 2021). These findings indicate that CeO_2_ nanoparticles are promising materials for further biotherapeutic research.

In this study, we designed and synthesized cerium oxide nanoparticles encapsulating Nrf2 plasmids (CeO_2_/Nrf2 nanocomposites) to synergistically target both the oxidative stress and inflammatory response, leading to an innovative and effective therapeutic strategy for myocardial infarction. We showed that the nanocomposites delivered Nrf2 plasmids to the heart. Furthermore, the CeO_2_/Nrf2 nanocomposites effectively attenuated cardiomyocyte apoptosis and cardiac dysfunction post myocardial infarction by reducing ROS and inflammation responses.

## 2 Materials and methods

### 2.1 Reagents

Cerium (IV) acetate (529,559) was purchased from Sigma-Aldrich (Shanghai) Trading Co. (Darmstadt, Germany). 1,2-Dioleoyl-3-trimethylammonium-propane (chloride salt) (DOTAP) (LP-R4-117) was purchased from Xi’an Ruixi biological Technology Co. (Xi’an, China). 1,2-Dioleoyl-sn-glycero-3-phosphoethanolamine (DOPE) (F20170000595) was purchased from AVT (Shanghai) Pharmaceutical Technology Co. (Shanghai, China). Dulbecco’s modified eagle medium (DMEM) (C11995500BT), DMEM/F-12 (C11330500BT), trypsin (25,200-072) and Penicillin Streptomycin (15140122) were obtained from Gibco (Grand Island, NY, United States). Fetal bovine serum (FBS) (FSP500) was purchased from Excell Bio (Jiangsu, China). Trizol lysis buffer (15596018) was obtained from Invitrogen Co. (California, United States). Phosphate buffered saline (PBS) (G0002-15), Hematoxylin and eosin (H&E) staining solution (G1076-500ML) and Masson’s trichrome staining solution (G1006) were purchased from Wuhan Servicebio Technology Co. (Wuhan, China). RIPA Lysis Buffer, reactive oxygen species assay kit (S0033S), cell counting kit-8 (C0038) and Nuclear and cytoplasmic protein extraction kit (P0028) were obtained from Beyotime Biotechnology (Shanghai, China). PolyJet™ *in vitro* DNA Transfection Reagent (SL100688) was purchased from SignaGen Laboratories (Maryland, United States). *In Situ* Cell Death Detection Kit (12156792910) was obtained from Roche Diagnostics Deutschland GmbH (Mannheim, Germany). Pierce™ BCA Protein Assay Kits (23,227), SuperSignal West Pico PLUS (34,580) and DNase I (18068015) were obtained from Thermo Fisher Scientific (Jiangsu, China). Dihydroethidium (DHE) (KGAF019, KeyGEN) was purchased from KeyGEN BioTECH (Nanjing, China). EndoFree Mini Plasmid Kit II (DP118-02) was purchased from TIANGEN Biotech Co. (Beijing, China). Anti-Nrf2 (E3J1V for WB), anti-GAPDH (14C10) and Cleaved-Caspase-3 antibody (9964S) were purchased from Cell Signaling Technology (Danvers, MA, United States). Anti-Nrf2 (ab31163 for Immunofluorescence), anti-Heme Oxygenase 1 antibody (ab68477) were obtained from Abcam (Cambridge, MA). Beta-tubulin (TA-10) was purchased from ZSGB-BIO (Wuxi, China). Lamin B1 Polyclonal Antibody (AP6001) was obtained from Bioworld Technology, Inc. (Shanghai, China). iNOS Polyclonal antibody (18985-1-AP) and HRP-conjugated secondary mouse or rabbit antibodies were obtained from Proteintech Group, Inc. (Chicago, United States). Anti-CD68 Antibody (137,001) was obtained from BioLegend (San Diego, CA). α-actinin (BM003) was obtained from Boster Biological Technology co. (California, United States). 4′,6-diamidino-2-phenylindole (DAPI) (C0065) was purchased from Beijing Solarbio Science & Technology Co. (Beijing, China). Fluorescent secondary antibody was obtained from Abbkine Biotechnology Co. (Wuhan, China). Sangon Biotech (Shanghai, China).

### 2.2 Preparation of the ceria nanoparticles and CeO_2_/Nrf2 nanocomposites

Briefly, oleamine (3.2 g) and cerium (IV) acetate hydrate (1.36 mM) were dissolved in the xylenes (15 mL) and stirred overnight at room temperature. Subsequently, the mixture was heated to 90°C and ddH_2_O (1 mL) was added into the mixture under vigorous stirring. The mixture was further aged was aged at 90°C for 3 hours. After cooling to room temperature, the ceria nanoparticles were precipitated by adding equal volume of ethanol and harvested by centrifugation. After washing with ethanol three times, the ceria materials were dispersed in ethanol for further use.

The X-ray diffraction (XRD, 40 kV, 40 mA, D8 advanced, Bruker, Germany) and X-ray photoelectron spectrum (XPS) were employed to analyze the element of the ceria material. The Al X-ray source was used in XPS, the tube voltage was 15 kV and the tube current was 12 mA, the diameter beam spot was 500 µm (Escalab 250xi, Thermo Scientific, United States).

To prepare the positive charged ceria nanoparticles, the ceria materials (6.0 mg), the DOTAP (30.0 mg) and DOPE (30 mg) Pharmaceutical Technology Co., Shanghai, China) were dispersed in the methanol (5 mL). And then, rotary evaporation was used to completely remove the solvent of this mixture. Subsequently, the materials were re-dissolved by deionized water (20 mL) and handled by high-pressure homogenization at 800 bar for 10 min. The positive charged ceria nanoparticles were obtained following dialysis in the deionized water. The morphology of the ceria nanoparticles was imaged by the transmission electron microscope (TEM), the voltage was 100 kV (JEM 2100F, JEOL, Japan).

The plasmid encoding Nrf2 was added into the ceria nanoparticle solution with a series of nanoparticle to plasmid ratios (μL/μg) (N/P ratios, 4, 5, 7, 10, 12, 15, 17, 20, 24, 30, 40, 60, 120) and incubated at room temperature for 0.5 h. The plasmid encoding Nrf2 was successfully wrapped into the ceria nanoparticles and the plasmid - loading ceria nanoparticles complex was formed. The size and the zeta potential of the CeO_2_/Nrf2 nanocomposites were analyzed by the dynamic light scattering (DLS) method (ZetaSizer, Malvern, United Kingdom). The agarose gel electrophoresis was employed to confirm the complexation. The naked plasmid and the complex with different N/P ratios were loaded, the voltage was 100 kV. The CeO_2_/Nrf2 nanocomposites with N/P ratio of 5 was stored in PBS (with 10% serum) at room temperature for up to 7 days, then its stability was assessed in the aspects of particle size.

### 2.3 Assessment of antioxidant activity

It was found that Ceria nanoparticles increased superoxide dismutase (SOD) and catalase activity. And this mimetic activity that has been assumed to be accountable for cellular defense by nanoceria. The effects of the ceria nanoparticle on the SOD activity were measured by commercial kit (Nanjing Jiancheng Bioengineering Institute, Jiangsu, China) according to instruction for authors. The ceria nanoparticles were used at different concentrations (0, 0.1, 0.2, 0.4, 1.0, 2.0, 4.0 µM).

The effects of the ceria nanoparticle on the catalase activity were further assayed by detecting the residual H_2_O_2_ amount. Briefly, different concentrations of the ceria nanoparticles (0, 15, 30, 60, 90, 120, 150 µM) were incubated with H_2_O_2_ (40 µM) at room temperature for 3 h. And then the horseradish peroxidase (HRP) and the TMB solution was further added into the mixture and incubated at 37°C for another 0.5 h. Sulfuric acid was used to terminate the reaction and the absorbance was measured at 450 nm (Epoch, Biotek, United States). Compared with the H_2_O_2_ standard curve, the residual H_2_O_2_ amount could be determined.

### 2.4 *In vivo* tracking study

To investigate the targetability of the CeO_2_/Nrf2 nanocomposites *in vivo*, we prepared DiR-abeled CeO_2_ nanoparticles and CeO_2_/Nrf2 nanocomposites for tracking study. The prepared nanoparticles were injected intravenously through the tail vein of C57BL/J6 mice. At 0.5–72 h after injection, images were taken using the IVIS Lumina imaging system. After *in vivo* tracking, the tissues (heart, lung, liver, kidneys, and spleen) of the mice were collected and subjected to *ex vivo* imaging (Liang et al., 2022).

### 2.5 Mice

In this study, all animal experiments were approved by the Animal Research Committee, Guangzhou Medical University (Guangzhou, China). All experiments were approved by the Institutional Animal Care and Use Committee, Guangzhou Medical University, Guangzhou, China (the approved ethic No. was 2019-590). Male C57BL/6J mice (9 weeks old, 20–25 g) were purchased from the Medical Experimental Animal Center of Guangdong Province and housed in conditions of temperature (23°C ± 2°C), humidity (60% ± 5%) and 12 h light-dark cycle at the Center of Laboratory Animal, Guangzhou Medical University. They were received humane care and free to food and water. All the experimental procedures were carried out according to the guidelines for the Care and Use of Laboratory Animals published by the United States National Institutes of Health (NIH Publication, revised 2011).

### 2.6 Mouse model of myocardial infarction and groups

Animal model of myocardial infarction was established through ligation of the left anterior descending artery (LAD) as we previously described (Zheng et al., 2020). Briefly, the mice were anesthetized by intraperitoneally administration with 4.5% pentobarbital hydrochloride (Merial, Hallbergmoos, Germany) and connected to a respirator for mechanical ventilation. After thoracotomy between the third and the fourth rib on the left, the heart was exposed and the LAD coronary artery was ligated by using 8–0 silk suture (Ningbo Medical Needle Co., Ningbo, China). Animals in the sham group underwent the same procedure but without ligation.

Total amount of 79 mice were randomized separated into 5 different groups: (1) the sham group (sham, n = 15); (2) myocardial infarction group (MI, n = 15); (3) MI + Nrf2 plasmid alone treated group (MI + Nrf2, n = 15); (4) MI + CeO_2_ nanoparticles alone treated group (MI + CeO_2_, n = 17); (5) MI + the complex of CeO_2_ nanoparticles with Nrf2 plasmid treated group (MI + CeO_2_/Nrf2 nanocomposites, n = 17). The equivalent volume of normal saline was used as the control solvent. The Nrf2 plasmid solvent (5 ug/10 g body weight), CeO_2_ nanoparticles (N/P 8:1) and the CeO_2_ nanoparticles with Nrf2 plasmid (5 ug/10 g body weight, N/P 8:1) were administrated separately to mice via tail vein at different time point (0, 24 h, 3 days, 5 days or 7 days) post MI surgery. Echocardiography was performed at the third day or the 14th day post MI.

### 2.7 Echocardiography assessment

As we previously described (Wu et al., 2022), transthoracic echocardiography was performed by using Vevo 2100 imaging system (Visual Sonics, Toronto, Canada) with high-frequency ultrasonography while the mice were awake. The left ventricle was viewed at short axis M-mode. The echocardiographic parameters of left ventricle such as left ventricular ejection fraction (LVEF), left ventricular fractional shortening (LVFS), left ventricular internal diastolic diameter (LVIDD), left ventricular internal systolic diameter (LVIDS), left ventricular end-diastolic anterior wall (LVAWD), left ventricular end-systolic anterior wall (LVAWS), left ventricular end-diastolic posterior wall (LVPWD), left ventricular end-systolic posterior wall (LVPWS) were measured.

### 2.8 Histological analysis

The heart tissues were fixed with 4% paraformaldehyde overnight at 4°C. Hematoxylin and eosin (H&E) staining and Masson’s trichrome staining (Wuhan Servicebio Technology Co., Wuhan, China) were performed as we described previously (Zheng et al., 2020; Wu et al., 2018).

For immunofluorescent (IF) (Wu et al., 2022), paraffin sections of myocardial samples were treated with primary Nrf2 (1:100, Abcam, Cambridge, MA) antibody overnight at 4°C. Then, samples were incubated with secondary DyLight 594-labelled Goat Anti-Rabbit IgG (1:250, a23320; Abbkine Biotechnology Co., Wuhan, China) at room temperature for 2 h. 4′,6′-diamidino-2-phenylindole (DAPI, 10 μg/mL) was used to stain the nuclei. Fluorescence was captured by confocal microscopy and were analyzed using ImageJ software.

### 2.9 Myocardial dihydroethidium (DHE) staining and TdT-mediated dUTP nick-end labeling (TUNEL) staining

DHE staining was performed as we previously described (Gao et al., 2022). Briefly, the fresh heart tissues were rapidly excised, fixed in 4% paraformaldehyde and embedded in OCT Compound (TISSUE-TEK; Sakura Finetek United States, Inc.). And then, 10 μm thick sections were cut and incubated with 10 mM DHE (KGAF019, KeyGEN) at 37°C for 30 min 4′,6′-diamidino-2-phenylindole (DAPI, 10 μg/mL) was used to indicate the nuclei. Images were obtained by fluorescent microscopy and were analyzed by ImageJ software.

TUNEL staining was preformed as we previously described (Zheng et al., 2020). Briefly, myocardial apoptosis was measured with *In Situ* Cell Death Detection Kit, TMR red (Roche Diagnostics Deutschland GmbH, Mannheim, Germany) according to manufacturer’s instruction. Images were attained by fluorescent microscope with 200x magnification and evaluated by ImageJ. The TUNEL positive stained cells were counted in 4 views in AAR and normalized to total area of the image determined by ImageJ.

### 2.10 Cell culture and hypoxia model

The H9C2 cardiac myoblasts were purchased from the American Type Culture Collection (ATCC, Rockville, MD) and were cultured in high-glucose (4.5 g/L) Dulbecco’s modified Eagle’s medium (DMEM; Gibco, Grand Island, NY, United States) supplemented with 10% fetal bovine serum (Excell Bio, Jiangsu, China) and 1% penicillin/streptomycin (Gibco, Grand Island, NY, United States) in an incubator containing 95% air and 5% CO_2_ at 37°C. Passage 3–9 of cells was used.

To mimic myocardial ischemia *in vitro*, H9C2 cardiac myoblasts were subjected to oxygen-glucose deprivation (OGD) as we previously described (Wu et al., 2018). The hypoxic/ischemia group was prepared by replacing the growth medium of the cells with serum-free dulbecco’s modified eagle medium nutrient mixture F-12(Ham) (Gibco, Grand Island, NY, United States), and then incubating the H9C2 cardiac myoblasts at 37°C in an incubator containing 94% N_2_, 5% CO_2_, and 1% O_2_ for 6 h. Control cells were kept in normal medium under normal conditions. To examine the effect of CeO_2_ nanomaterials and CeO_2_/Nrf2 nanocomposites on OGD-induced cardiomyocyte injury, H9C2 cardiac myoblasts were treated with or without CeO_2_ nanomaterials for 18 h followed by OGD for 6 h before the next experiments. all the experiments were repeated 3 times.

RAW264.7 macrophages were obtained from American Type Culture Collection (ATCC, Rockville, MD), and cultured in high-glucose (4.5 g/L) Dulbecco’s modified Eagle’s medium supplemented with 10% fetal bovine serum (Excell Bio) and 1% penicillin/streptomycin in an incubator containing 95% air and 5% CO_2_ at 37°C.

### 2.11 Plasmid transfection

For DNA transfection (Chuang et al., 2014), the NFE2L2 plasmid (NM_006164) (Beijing Tsingke Biotech Co.) were transfected into H9C2 cardiac myoblasts according to the manufacturer’s instructions. Cells were plated 24 h prior to transfection. For each well of a 6-well plate, 2 µg of DNA was diluted into 100 µL of serum-free DMEM with high glucose. Additionally, 6 µL of PolyJet™ reagent (SignaGen Laboratories, Maryland United States) was diluted into 100 µL of serum-free DMEM with high glucose. The diluted PolyJet™ reagent was immediately added to the diluted DNA solution all at once. The solution was vortex-mixed and incubated for 10–15 min at room temperature to allow transfection complexes to form. At the end of incubation, 2.0 mL of pre-warmed complete cell growth medium was added to the cells and plated onto a well of a 6-well plate. The cells were incubated at 37°C with 5% CO_2_. The transfection complex-containing medium was gently removed and replaced with complete culture medium 12–16 h after plating. Transfection efficiency was checked 48 h post-transfection.

### 2.12 Cell viability assay

H9C2 cardiac myoblasts were seeded in 96-well plates at 6 × 10^3^ cells per well and incubated for 24 h. Subsequently, the cells were treated with varying concentrations of CeO_2_ nanoparticles. As we have described previously (Gao et al., 2022), Cell viability was assessed using the CCK-8 assay kit (Beyotime Biotechnology, Shanghai, China) according to the manufacturer’s protocol. Briefly, 10 µL of CCK-8 reagent was added to each well containing culture medium, followed by incubation at 37°C in the dark for 1 h. Absorbance of each well was measured at 450 nm using a spectrophotometer. Furthermore, to evaluate cell viability of the different treatment groups, the CCK-8 assay was conducted again after hypoxia exposure.

### 2.13 DCFH-DA assay

Intracellular ROS levels were assessed using DCFH-DA probe (Beyotime Biotechnology, Shanghai, China) according to the manufacturer’s instructions. Briefly, the cells were treated with DCFH-DA (10 μM) for 20 min at 37°C. After incubation, the cells were washed for 3 times and then Medium was added for microscopic imaging.

Images were acquired with Nikon A1 (Nikon America Inc., Melville, NY) confocal microscope and the intensity was measured by ImageJ software. For quantification, 3 images of different views were taken blindly and the green fluorescence intensity was counted by ImageJ. Representative images/figures were selected according to their quality and to most accurately represent the group mean/average across all the available data.

### 2.14 Western blot

As we described previously (Zheng et al., 2020), the tissue of the infarct hearts was separated and homogenized, the nucleus and cytoplasmic fractions of treated cells were collected by a Nuclear and Cytoplasmic Extraction Reagents kit (Beyotime, Shanghai, China). The protein concentration was determined using the BCA Protein Assay Reagent Kit (Thermo Fisher Scientific, Jiangsu, China). Protein samples were separated by electrophoresis using a 10% SDS-PAGE gel and transferred to polyvinylidene difluoride (PVDF) membrane (Millipore, United States). The PVDF membrane was blocked and incubated with the primary antibody overnight at 4°C. And then, the membrane was further incubated with secondary antibody for 1 h at room temperature. Blots were developed using the SuperSignal West Pico PLUS (Thermo Fisher Scientific, Jiangsu, China). The band intensity was analyzed with ImageJ software.

### 2.15 Quantitative real-time PCR (qPCR)

Myocardial tissues or H9C2 cardiac myoblasts were homogenized in TRIzol (1 mL, Invitrogen, California, United States) to extract total RNA. The cDNA was amplified by using AG RNAex Pro Reagent (Accurate Biology, Hunan, China) and qPCR was performed as we described previously (Zheng et al., 2020). Primer sequences were listed in Supplementary material online (Supplementary Table S1).

### 2.16 Statistical analysis

All data was analyzed with GraphPad Prism, version 8.0 (GraphPad Software, Boston, MA, United States) and shown as means ± SEM. Difference between two groups were analyzed using Student’s *t*-test. For three or more groups, the normal distribution was confirmed by the Shapiro–Wilk test (*P* > 0.1) with SPSS 17.0. Differences among three or more groups were analysed using one-way ANOVA. Statistical significance was accepted for *P* < 0.05. Further Tukey *post hoc* analysis (α = 0.05) was performed to confirm where the differences occurred between groups. The detailed *P*-value compared with respective controls is presented in the corresponding Figure.

## 3 Results

### 3.1 Characterizations of the ceria nanoparticles

**FIGURE 1:**
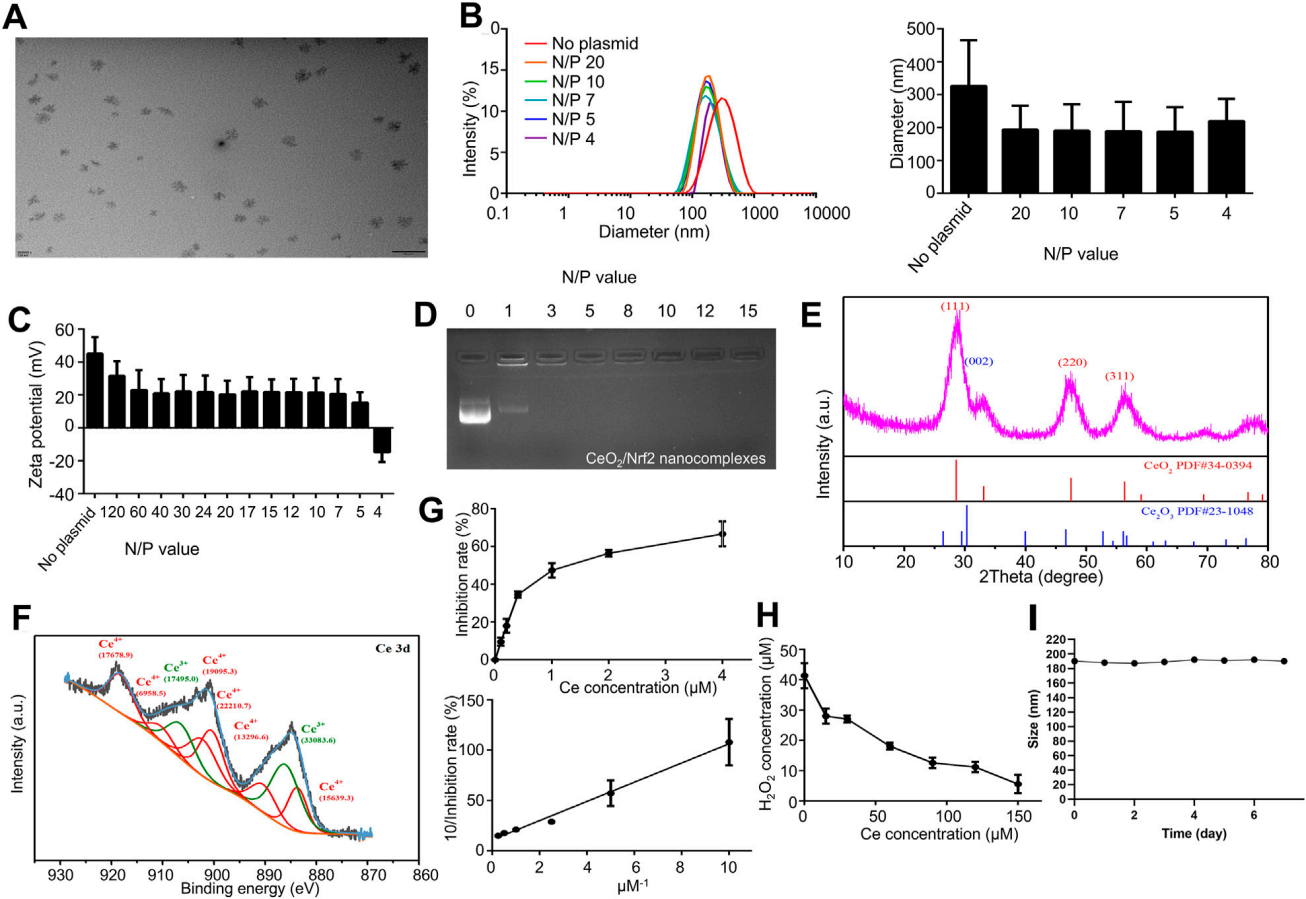
Preparation and Characterization of CeO_2_/Nrf2 nanocomposites **(A)** Representative TEM images of ceria nanoparticles. **(B)** The diameter and **(C)** zeta potentials of ceria nanoparticles with or without plasmid Nrf2 at different N/P ratio. **(D)** Electrophoretic mobility retardation assay of CeO_2_/Nrf2 nanocomposites at various N/P ratios. **(E)** The XRD and **(F)** XPS spectrum of the ceria nanoparticles. **(G)** The SOD activity and **(H)** the catalase activity of ceria nanoparticles. **(I)** The particle size of CeO_2_/Nrf2 nanocomposites with N/P value of 5 in the serum solution at room temperature.

Ceria nanoparticles were synthesized using a previously published simple wet chemistry method (Li et al., 2022). The morphology of the ceria nanoparticles was observed using transmission electron microscope (TEM). As shown in Figure 1A, the ceria nanoparticles were well-dispersed without significant agglomeration, with an average diameter of approximately 30 nm. Further analysis of the size and zeta potential of the ceria nanoparticles was conducted. Dynamic light scattering (DLS) results showed an average nanoparticle size of 326.8 ± 138.9 nm (PDI: 0.271) and a zeta potential of 45.2 ± 9.9 mV (Figures 1B, C).

After loading with Nrf2 plasmids, the nanoparticle size decreased to approximately 190.7 ± 80.3 nm, and the zeta potential decreased with increasing nucleic acid ratios. Specifically, the zeta potential declined to 15.4 ± 6.2 mV at an N/P ratio of 5. The CeO_2_/Nrf2 nanoparticles were negatively charged at an N/P ratio of 4, indicating plasmid overload. The reduction in particle size and positive zeta potential confirmed the successful condensation of the ceria nanoparticles with the nucleic acid, as further validated by agarose gel electrophoresis (Figure 1D). The optimized N/P ratio of 5 indicated that the ceria nanoparticles had a promising nucleic acid loading capacity. In addition, the particle size measurements indicated that the CeO_2_/Nrf2 nanocomposites had good stability (Figure 1I).

Subsequently, X-ray diffraction (XRD) and X-ray photoelectron spectroscopy (XPS) were employed to determine the valence states of cerium in the nanoparticles. In the XRD spectrum, the (002) peak was attributed to Ce^3+^, while the (111), (220), and (311) peaks were attributed to Ce^4+^ (Figure 1E). The Ce 3 days spectrum was deconvoluted into two groups of peaks: 883.80, 891.15, 900.75, 903.00, 912.30, 918.75 eV corresponding to Ce^4+^, and 886.40, 907.40 eV corresponding to Ce^3+^. The results indicated that both Ce^3+^ and Ce^4+^ co-exist in the ceria nanoparticles, with a ratio of approximately 1:1.9 (Ce^3+^/Ce^4+^) (Figure 1F).

The transition between Ce^3+^ and Ce^4+^ is believed to facilitate the elimination of ROS. To comprehensively assess the antioxidant capacity of the synthesized cerium nanoparticles, free radical scavenging experiments were conducted at varying concentrations. The superoxide dismutase (SOD) and catalase mimetic activities of the ceria nanoparticles were measured. The ceria nanoparticles were capable of eliminating superoxide anions and reducing absorbance at 560 nm. As shown in Figure 1G, the SOD mimetic activity of the ceria nanoparticles increased proportionally with the concentration of ceria. The linear relationship between the 1/inhibition rate and the 1/ceria concentration further validated this observation. Similarly, the ceria nanoparticles effectively eliminated H_2_O_2_ in a concentration-dependent manner as demonstrated in Figure 1H. Additionally, the cytotoxicity of the ceria nanoparticles was assessed using H9C2 cardiac myoblasts. As shown in Supplementary Figure S1A, CeO_2_ nanoparticles exhibited no significant cytotoxicity in H9C2 cardiac myoblasts. These findings indicate that the ceria nanoparticles and CeO_2_/Nrf2 nanocomposites possess SOD and catalase mimetic activities without cytotoxic effects.

### 3.2 *In Vitro* cellular uptake and *in vivo* distribution of CeO_2_/Nrf2 nanocomposites

**FIGURE 2:**
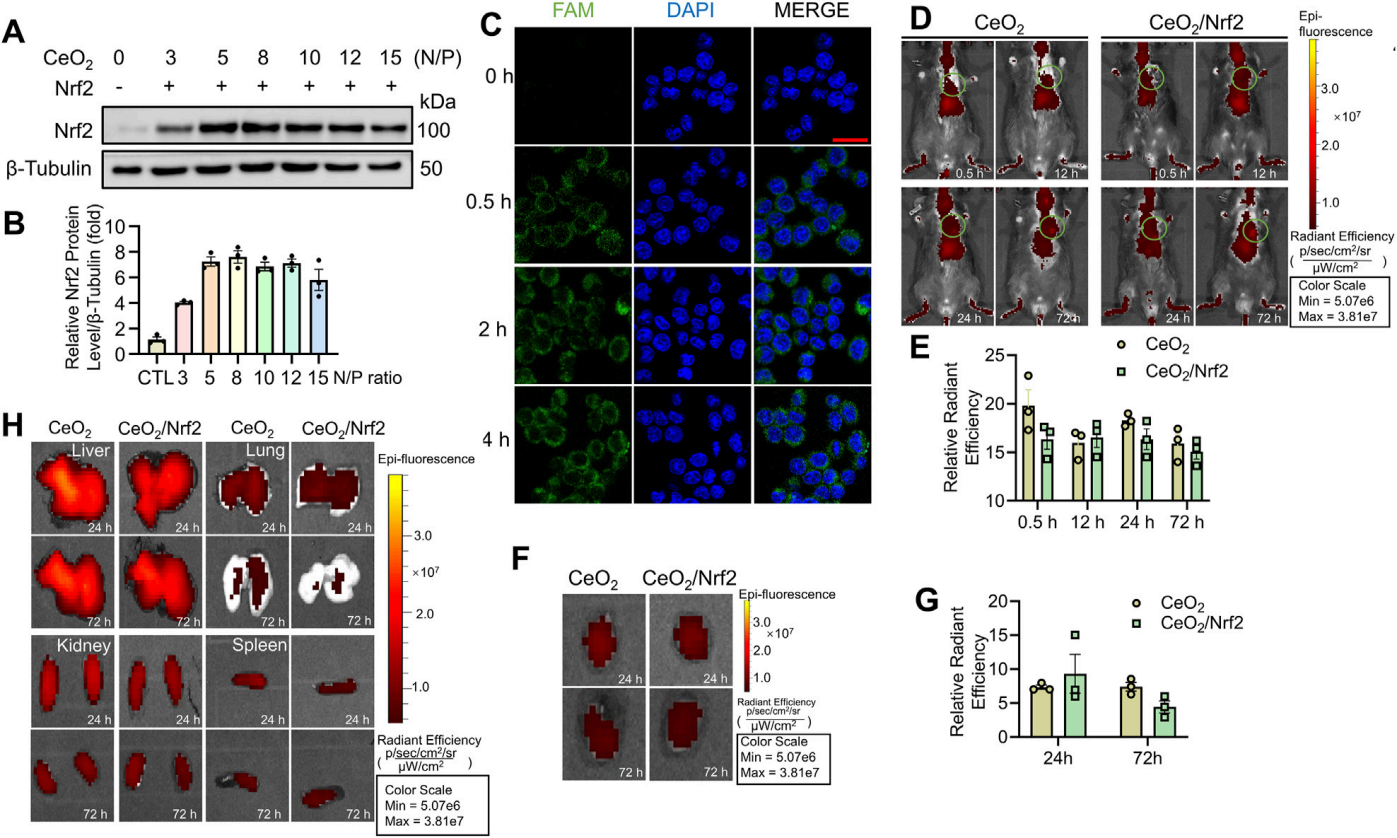
The *in vitro* cellular uptake and *in vivo* distribution of CeO_2_/Nrf2 nanocomposites **(A)** Representative immunoblotting image and **(B)** quantification of Nrf2 protein level in H9C2 cardiac myoblasts treated with different ratios of CeO_2_ nanoparticles to Nrf2 plasmids (n = 3). **(C)** CeO_2_/FAM–labelled Nrf2 nanocomposites (green) was incubated with macrophage for different time periods at the N/P ratio of 8. Representative confocal fluorescence images of CeO_2_/Nrf2 nanocomposites in macrophage were measured (scale bar, 20 µm). **(D)** After tail injection to mice, *in vivo* accumulation and retention of DiR-labled ceria nanoparticles or CeO_2_/Nrf2 nanocomposites were detected at different time points via using Xenogen IVIS imaging system, the circle indicates the position of the heart and **(E)** the radiant efficiency was quantified (n = 3). **(F)** Accumulation of DiR-labled ceria nanoparticle or CeO_2_/Nrf2 nanocomposites in the isolated mouse heart at different time points after tail injection and **(G)** the quantification (n = 3). **(H)** The accumulation of the ceria nanoparticle or CeO_2_/Nrf2 nanocomposites in the liver, lung, kidney and spleen at different time points after tail injection.

Efficient cellular internalization is essential for effective gene transfection (Wang et al., 2018). Initially, Nrf2 plasmids were successfully transfected and expressed in H9C2 cardiac myoblasts using Lipofectamine 2000 (Supplementary Figure S2A). Subsequently, CeO_2_/Nrf2 nanocomposites were prepared at various N/P ratios and incubated with H9C2 cardiac myoblasts. As shown in Figures 2A, B, Nrf2 expression was significantly elevated, reaching a peak at an N/P ratio of 8, indicating that CeO_2_ nanomaterials can efficiently transfect Nrf2 plasmids into cardiomyocytes. The optimal N/P ratio for CeO_2_/Nrf2 nanocomposites was determined to be 8. Furthermore, the successful uptake of the Nrf2 plasmid was further confirmed by labeling the plasmid with the fluorescent dye FAM. Macrophages were incubated with CeO_2_/FAM-labeled Nrf2 nanocomposites for different durations (0 h, 0.5 h, 2 h, and 4 h). As shown in Figure 2C, the CeO_2_/Nrf2 nanocomposites were rapidly internalized by macrophages within 0.5 h and localized around the nucleus.

To monitor the distribution and targeting ability of the CeO_2_/Nrf2 nanocomposites to the cardiac infarction zone, a mouse model was established via permanent ligation of the left anterior descending artery. The CeO_2_/Nrf2 nanocomposites or CeO_2_ nanomaterials were administered to mice via tail vein injection within 0.5-to-1-h post-MI. As shown in Figures 2D, E, both CeO_2_ nanomaterials and CeO_2_/Nrf2 nanocomposites distributed throughout the entire body within 72 h after injection, with a strong signal observed in the heart. At 24- and 72-h post-injection, the organs (heart, lung, liver, kidneys, and spleen) were isolated. Significant accumulation of CeO_2_ nanomaterials and CeO_2_/Nrf2 nanocomposites in the heart was observed after 24 h (Figures 2F, G). This CeO_2_/Nrf2 nanocomposite-based delivery system, administered via tail vein injection, demonstrated effective accumulation in the heart. Distribution in other organs was also detected (Figure 2H). Moreover, H&E staining results showed that CeO_2_/Nrf2 nanocomposites did not alter the histological features of these organs (Supplementary Figure S2B). These findings indicate that cerium oxide nanomaterials facilitate the accumulation and retention of Nrf2 in the heart with myocardial infarction.

### 3.3 Delivery of Nrf2 via ceria nanoparticles in a mice model of MI and its biological performance

**FIGURE 3:**
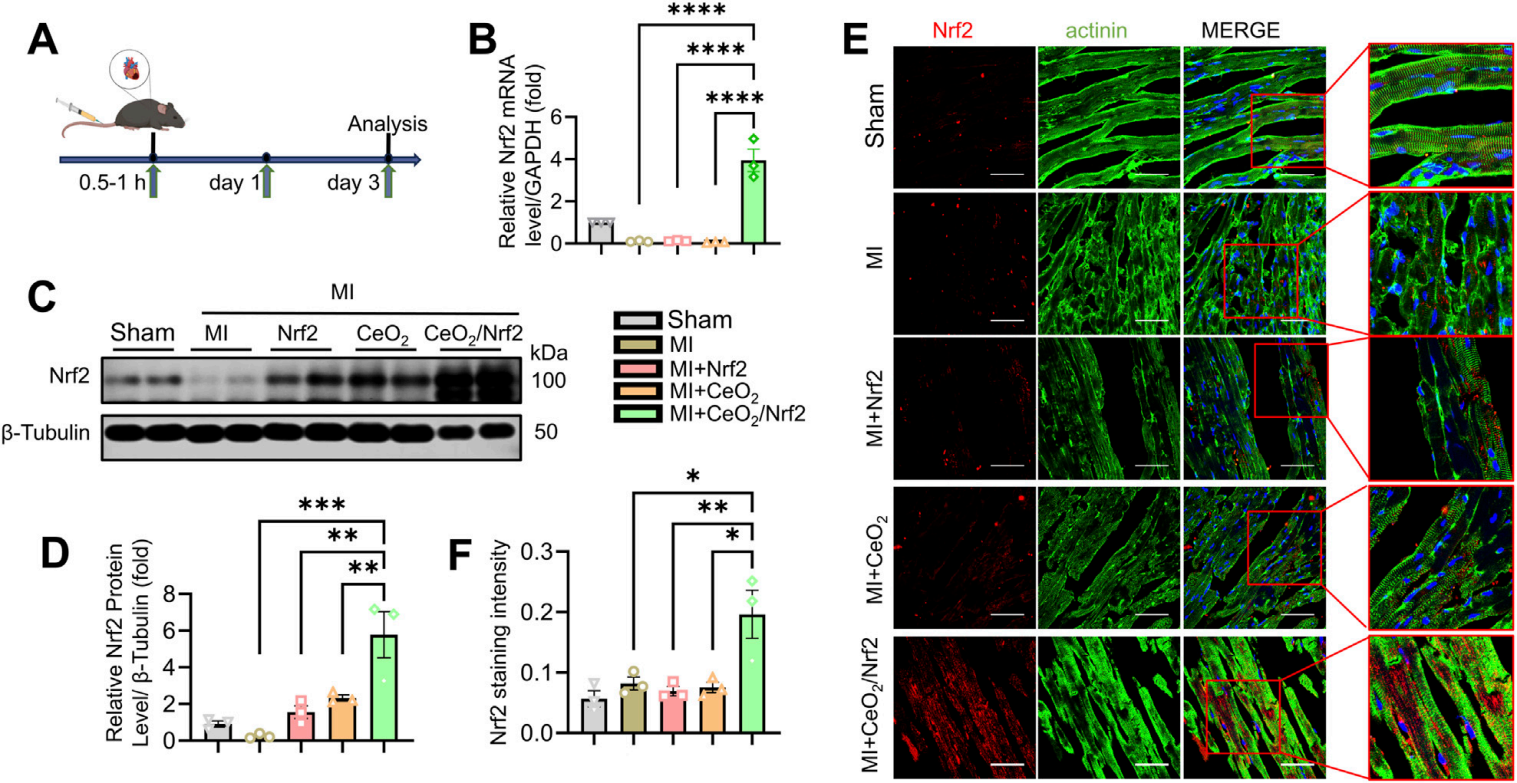
Delivery of Nrf2 via Ceria nanoparticles in myocardial infarction mice model and its biological performance **(A)** The scheme of experiment. **(B)** The mRNA level of Nrf2 were measured by qPCR in different groups (n = 3). GAPDH acted as a home keeper gene. **(C)** Representative immunoblotting image and **(D)** quantification of the protein level of Nrf2 in the heart tissue (n = 3). **(E)** Immunofluorescence staining Nrf2 expression (red) and the maker of cytoskeleton actinin (green) in heart tissue (scale bars, 50 μm). **(F)** The quantification based on immunofluorescence intensity of Nrf2 in **(E)** using ImageJ software (n = 3). Data are shown as the mean ± SEM, ^*^ *p* < 0.05, ^**^ *p* < 0.01, ^***^ *p* < 0.001 and ^****^ *p* < 0.0001, n = 3 independent experiments.

The objective of this study was to deliver Nrf2, a key regulator during oxidative stress, to the heart using ceria nanoparticles. To validate the *in vivo* delivery efficacy of ceria nanoparticles for Nrf2, we prepared Nrf2 plasmid, CeO_2_ nanoparticles, and CeO_2_/Nrf2 nanocomposites, which were administered to mice via tail vein injection within 0.5–1 h post-MI surgery, with repeated doses on the first and third days post-MI (Figure 3A). Compared to the groups treated with Nrf2 plasmid or CeO_2_ nanoparticles alone, the mRNA and protein levels of Nrf2 were significantly higher following treatment with CeO_2_/Nrf2 nanocomposites (Figures 3B–D). Additionally, immunofluorescence results indicated that the red fluorescence intensity of Nrf2 was stronger in the CeO_2_/Nrf2 nanocomposite-treated group compared to the groups treated with Nrf2 plasmid alone or ceria nanoparticles alone (Figures 3E, F). These results consistently demonstrated that CeO_2_/Nrf2 nanocomposites effectively delivered Nrf2 into the heart tissue in a myocardial infarction model.

### 3.4 CeO_2_/Nrf2 nanocomposites ameliorated cardiomyocyte apoptosis and cardiac fibrosis post MI

**FIGURE 4:**
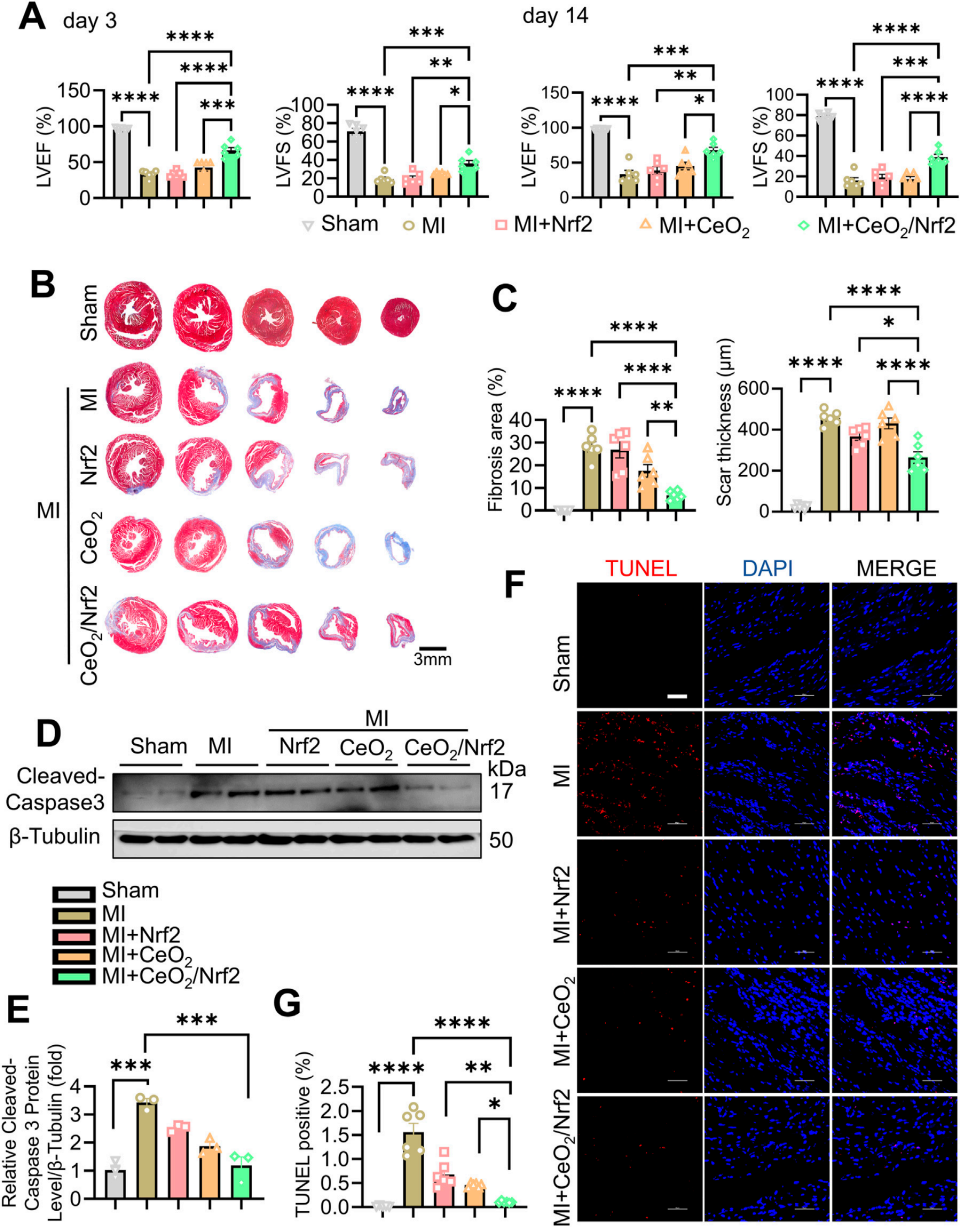
CeO_2_/Nrf2 nanocomposites improved myocardial function and ameliorated fibrosis post MI Mice were treated with ceria nanoparticles, Nrf2 plasmid or CeO_2_/Nrf2 nanocomposites by tail injection post MI. Cardiac function of left ventricular were measured by Echocardiographic analysis at day 3 and day 14 post MI. **(A)** Left ventricular ejection fraction (LVEF) and left ventricular fractional shortening (LVFS). n = 6 from independent experiments. **(B)** Representative images of Masson’s Trichrome staining of the heart. **(C)** Fibrosis area and scar thickness of the heart tissue were quantified post MI for 14 days (scale bar, 3 mm). n = 6 mice. **(D)** Representative immunoblotting image and **(E)** quantification of the Cleaved-caspase3 protein level. n = 3. **(F)** Representative images and **(G)** quantification of TUNEL staining from the heart tissue (scale bar, 50 μm). n = 6 mices. Data were shown as mean ± SEM, ^*^ *p* < 0.05, ^**^ *p* < 0.01, ^***^ *p* < 0.001 and ^****^ *p* < 0.0001.

CeO_2_/Nrf2 nanocomposites, along with Nrf2 plasmid and CeO_2_ nanomaterials, were prepared and administered to mice post MI via tail vein injection within 0.5–1 h post-MI surgery. The administrations were repeated on days 1, 3, 5, and 7. Cardiac function was assessed using transthoracic echocardiography on days 3 and 14 post-MI (Supplementary Figure S3A; Supplementary Table S2). As expected, left ventricular ejection fraction (LVEF) and left ventricular fractional shortening (LVFS) were significantly reduced, while left ventricular internal diameter (LVID) was increased at days 3 and 14 post-MI (Figure 4A; Supplementary Figure S3B; Supplementary Table S2). Treatment with CeO_2_ nanoparticles alone or CeO_2_/Nrf2 nanocomposites slightly improved these parameters. The CeO_2_/Nrf2 nanocomposites group exhibited superior cardioprotective effects compared to the Nrf2 plasmid or CeO_2_ nanoparticles alone groups. Masson trichrome staining was used to analyze cardiac fibrosis and collagen deposition post-MI. The results showed that fibrosis and collagen deposition were significantly alleviated in the CeO_2_/Nrf2 nanocomposite-treated mice compared to those treated with vehicle, Nrf2 plasmid alone, or CeO_2_ nanoparticles alone (Figures 4B, C). In summary, these findings suggest that CeO_2_/Nrf2 nanocomposites protect against MI-induced cardiac dysfunction and pathological remodeling in mice.

Apoptotic cell death of cardiomyocytes is a critical mechanism contributing to irreversible damage in MI (Chiong et al., 2011; Sheng et al., 2023). The protein level of Cleaved caspase-3, a key apoptosis effector, increased post-MI and was reduced by treatment with CeO_2_/Nrf2 nanocomposites or CeO_2_ nanoparticles alone (Figures 4D, E). Compared to the sham group, there was a significant increase in TUNEL-positive cells in the MI group, which was significantly reduced by treatment with CeO_2_/Nrf2 nanocomposites or CeO_2_ nanoparticles alone (Figures 4F, G). Additionally, CeO_2_/Nrf2 nanocomposites demonstrated a stronger protective effect against cardiomyocyte apoptosis than CeO_2_ nanoparticles alone.

### 3.5 CeO_2_/Nrf2 nanocomposites mitigated inflammation and ROS

**FIGURE 5:**
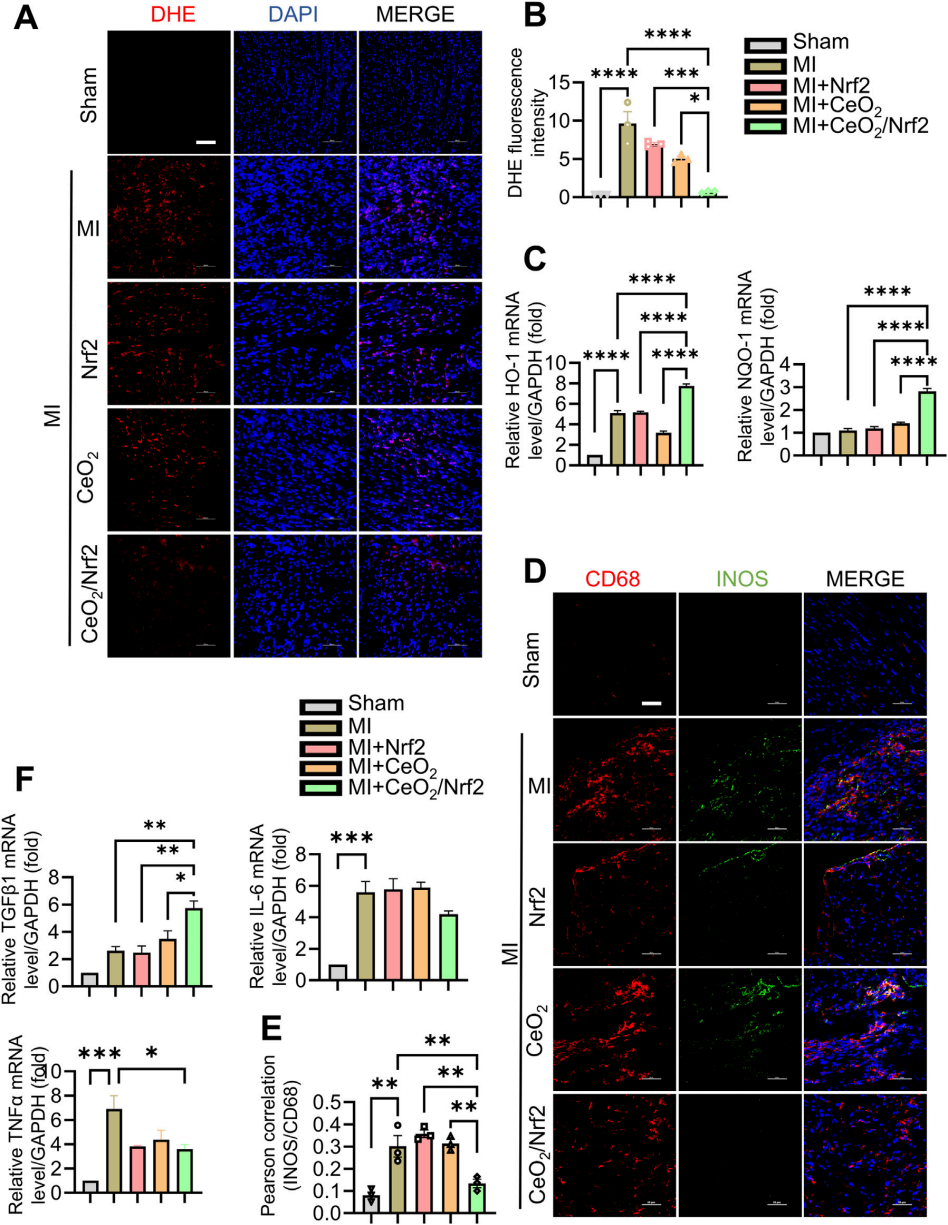
CeO_2_/Nrf2 nanocomposites mitigated inflammation and ROS **(A)** The level of ROS in cardiac sections of mice with MI for 3 days was measured by dihydroethidium (DHE) staining and the representative images (scale bar, 100 μm). **(B)** Quantification of the fluorescence intensity of DHE staining in **(A)**. **(C)** The mRNA levels of heme oxygenase 1 (HO-1) and NAD(P)H dehydrogenase quinone 1 (NQO1), n = 3. **(D)** Immunofluorescence staining CD68 expression (red) and M1 macrophage marker iNOS (green) on cardiac specimens from mice with MI (scale bar, 50 μm). **(E)** Quantification of the co-localization between iNOS and CD68 by using ImageJ software, n = 3. **(F)** The mRNA levels of TNFα, TGFβ and IL6, n = 3. Data were shown as mean ± SEM, ^*^ *p* < 0.05, ^**^ *p* < 0.01, ^***^ *p* < 0.001 and ^****^ *p* < 0.0001.

The surge of oxidative stress and inflammatory responses in the early stages post MI plays a crucial role in the progression of ischemic myocardial injury (Xiang et al., 2021). Nrf2, a dominant transcriptional factor in cellular defense against oxidative and xenobiotic stresses, activates downstream target genes such as heme oxygenase 1 (HO-1, also known as HMOX1) and NAD(P)H dehydrogenase quinone 1 (NQO1) (Wu et al., 2022; Mohs et al., 2021). To explore the protective mechanism of CeO_2_/Nrf2 nanocomposites, the level of intracellular ROS accumulation in the infarcted area was measured. DHE assays showed a burst of ROS production in the heart 3 days post-MI (Figures 5A, B). While treatments with Nrf2 plasmid and CeO_2_ nanoparticles alleviated ROS production, CeO_2_/Nrf2 nanocomposites exhibited stronger effects (Figures 5A, B). Furthermore, the mRNA levels of HO-1 and NQO1 were significantly elevated with CeO_2_/Nrf2 nanocomposites treatment in mice 3 days post-MI (Figure 5C), confirming the activation of Nrf2 defense mechanisms.

The interaction between oxidative stress and inflammation contributes to the progression and prognosis of MI (Wang et al., 2018). Therefore, the effects of CeO_2_/Nrf2 nanocomposites on inflammatory responses post-MI were further investigated. Immunohistochemical staining showed that CD68 (+) macrophages infiltrated the infarcted area 3 days post-MI, which was significantly reduced following CeO_2_/Nrf2 nanocomposites treatment (Figures 5D, E). The fluorescence intensity of iNOS, a marker of M1 macrophages, positively correlated with CD68 (+) indicated that CeO_2_/Nrf2 nanoparticles reduced M1 macrophages, suggesting their role in mitigating acute inflammatory responses. On the other side, there is no significant difference on M2 macrophages in the heart of different groups as evidence by co-localization of CD68 and Arg1 (a marker for M2 macrophages) (Supplementary Figure S4). Moreover, the mRNA levels of pro-inflammatory genes were elevated, and those of anti-inflammatory factors (TGFβ1, IL-6, and TNFα) were reduced 3 days post-MI. CeO_2_/Nrf2 nanocomposites treatment significantly ameliorated these MI-induced abnormal gene expression changes (Figure 5F). These results strongly indicate the antioxidant and anti-inflammatory properties of CeO_2_/Nrf2 nanocomposites during MI.

### 3.6 CeO_2_/Nrf2 nanocomposites protected H9C2 cardiac myoblasts against apoptosis and oxidative stress *in vitro*

**FIGURE 6:**
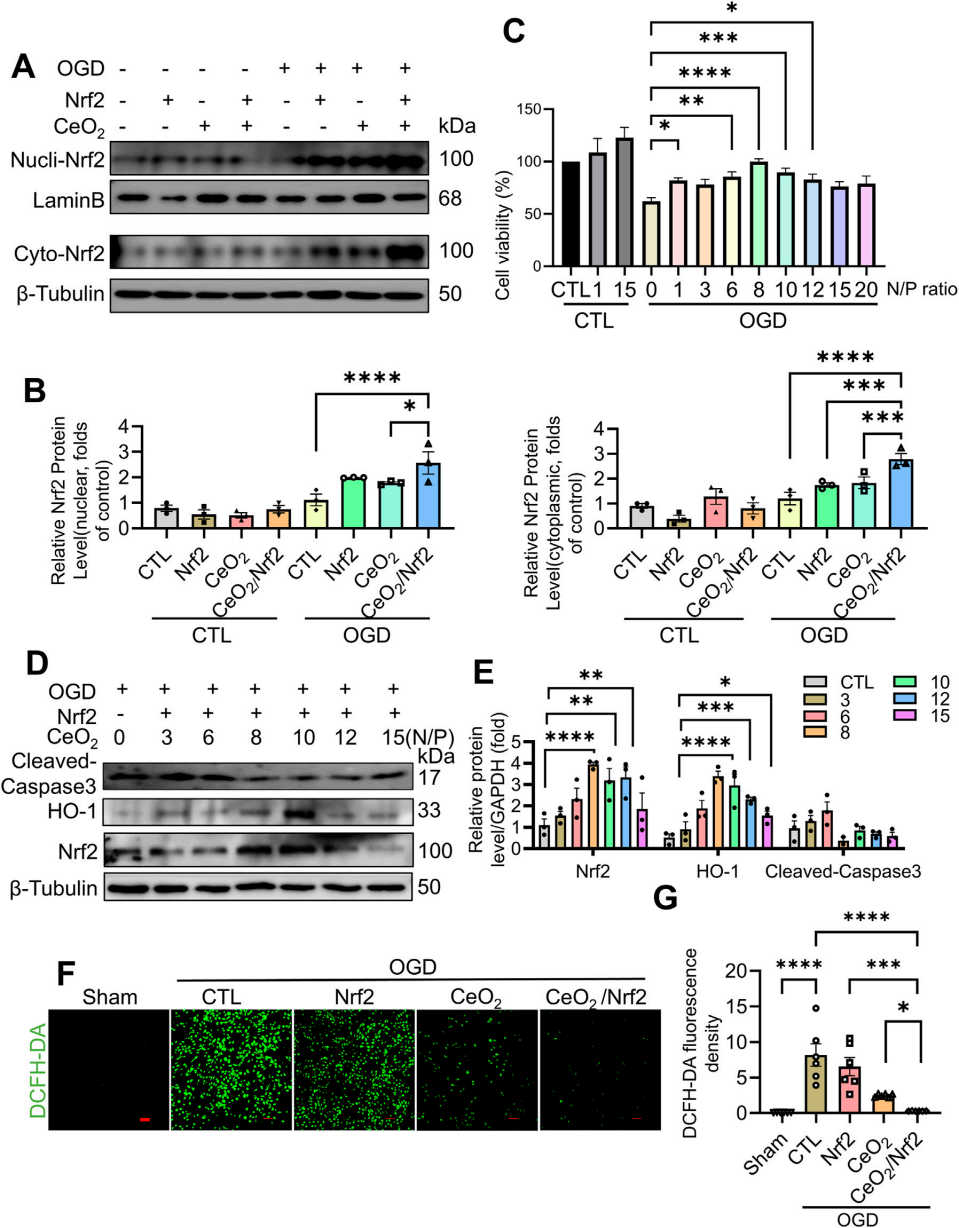
CeO_2_/Nrf2 nanocomposites protected against OGD-induced H9C2 cardiac myoblasts injury H9C2 cardiac myoblasts were subjected to OGD and transfected or treated with Nrf2 plasmid, CeO_2_ nanomaterials or CeO_2_/Nrf2 nanocomposites. Nucleus and cytoplasmic proteins were separated. **(A)** Representative immunoblotting image of Nrf2 protein. **(B)** Quantification of the protein level of Nrf2 in the nucleus and cytoplasm, n = 3. **(C)** H9C2 cardiac myoblasts were treated with CeO_2_/Nrf2 nanocomposites at different N/P ratios with or without OGD stimulation. Cell viability was measured by CCK8, n = 3. **(D)** Representative immunoblotting images of Nrf2, HO-1 and Cleaved-caspase3 protein. **(E)** Quantification of these proteins in **(D)**. H9C2 cardiac myoblasts were transfected with Nrf2 plasmids or treated with ceria nanoparticles, and suffered from OGD stimulation for 6 h **(F, G)** The ROS level was measured by DCFH-DA staining (scale bar, 100 μm) and the fluorescence intensity was quantified by using ImageJ software, n = 6. Data were shown as mean ± SEM, ^*^ *p* < 0.05, ^**^ *p* < 0.01, ^***^ *p* < 0.001 and ^****^ *p* < 0.0001.

To further elucidate the protective effects of CeO_2_/Nrf2 nanocomposites in cardiomyocytes, H9C2 cardiac myoblasts were used and subjected to oxygen-glucose deprivation (OGD) stimulation to mimic *in vivo* myocardial ischemia. The protein expressions of Nrf2, in both the nuclei and the cytoplasm, were significantly increased by CeO₂/Nrf2 nanocomposites, indicating effective delivery of Nrf2 plasmid into cardiomyocytes under OGD stimulation for 6 h (Figures 6A, B). Cell viability assays showed that CeO_2_/Nrf2 nanocomposites (at different N/P ratios) significantly improved the viability of cardiomyocytes under OGD stimulation (Figure 6C). Furthermore, the incubation of CeO_2_/Nrf2 nanocomposites with cardiomyocytes under OGD conditions at different N/P ratios significantly increased the protein levels of Nrf2 and HO-1, while decreasing the protein level of Cleaved-caspase3 (Figures 6D, E). Consistent with our previous results, the optimal N/P ratio for CeO_2_/Nrf2 nanocomposites was determined to be 8.

The DCFH-DA staining results indicated that oxidative stress levels in H9C2 cardiac myoblasts were elevated following OGD stimulation. Compared to the groups treated with either Nrf2 plasmid transfection or ceria nanoparticles alone, CeO_2_/Nrf2 nanocomposites exhibited a stronger effect in reducing ROS production in cardiomyocytes under OGD stimulation (Figures 6F, G). Consistent with our *in vivo* findings, CeO_2_/Nrf2 nanocomposites effectively protected cardiomyocytes from injury by elevating Nrf2 expression.

## 4 Discussion

In this study, we developed novel ceria nanoparticles encapsulating Nrf2 plasmids (CeO_2_/Nrf2 nanocomposites), which could be taken up by macrophages and delivered to the heart post-MI, without cytotoxicity to other organs. The CeO_2_/Nrf2 nanocomposites demonstrated superior effects in mitigating cardiac dysfunction, reducing infarct size, and attenuating cardiac remodeling post-MI compared to pristine ceria nanoparticles. Our results indicated that CeO_2_/Nrf2 nanocomposites significantly ameliorated *in vivo* and *in vitro* ROS generation and inflammatory responses by increasing the expression of Nrf2 and its target genes, subsequently reducing cardiomyocyte apoptosis.

Despite significant advances in the treatment of coronary artery disease and acute myocardial infarction (MI) over the past 2 decades, MI remains the leading cause of heart failure (Rallidis et al., 2022). Various strategies, such as thrombolysis therapy, percutaneous coronary intervention, and coronary artery bypass grafting, have reduced patient mortality; however, complications such as hemorrhage, ischemia-reperfusion injury, and coronary restenosis occur unpredictably (Mackman et al., 2020; Capodanno et al., 2022; Gershlick et al., 2013). Post-MI, therapeutic agents including drugs, mRNAs, and proteins cannot reach the myocardium with satisfactory efficiency, making drug delivery a primary challenge that impedes the development of cardiovascular therapy. Therefore, it is imperative to explore more efficient approaches to preserve myocardial function and prevent the progression to heart failure.

After MI, the dying cardiomyocytes triggering a powerful inflammatory response which is largely considered by inflammatory cell infiltration, particularly monocytes/macrophage (Lavine et al., 2018; Seropian et al., 2014). Especially, in the 48 h after AMI, macrophage is mobilized from the bone marrow and recruited infiltrate into the infarct. Therefore, this unique property makes macrophages an appealing vehicle for noninvasive imaging and targeted drug delivery to the infarcted myocardium (Chen et al., 2023). The nanoparticles, especially the positive-charged nanoparticles, such as the CeO_2_/Nrf2 nanocomposites in the research could be easily uptake by the macrophages (Figure 2C), and finally accumulated in the infarct. In this study, we observed that CeO_2_ nanoparticles delivered Nrf2 plasmids to monocytes and macrophages in the blood, spleen, and MI-affected heart. This indicates that CeO_2_ nanoparticles deliver their cargo to monocytes prior to their recruitment to the infarcted myocardium. Subsequently, the differentiated macrophages localize to the wound site to clear necrotic debris, facilitating the release of CeO_2_/Nrf2 nanoparticles in the infarct area.

ROS production and inflammatory responses are critically involved in the pathogenesis of MI and play a key role in the progression from MI to heart failure (Cheng et al., 2015). The detrimental effects of ROS are evidenced by findings that infarct size is significantly reduced in transgenic mice overexpressing superoxide dismutase (Hu et al., 2023; Chen et al., 1998). During MI, inflammatory cytokines such as tumor necrosis factor-α (TNF-α), IL-1β, and IL-6 are produced as part of the host response (Pearce et al., 2023). Excessive ROS further exacerbate inflammation, creating a vicious cycle that worsens myocardial injury by inducing additional ROS production (Zhao et al., 2022). However, the use of many antioxidant and anti-inflammatory drugs is limited by their short half-life, low stability, poor bioavailability, and side effects in treating MI (Liang et al., 2022; Naganuma and Traversa, 2012). Therefore, developing effective drugs and technologies to address these limitations is imperative (Saeed et al., 2023; Lozano et al., 2018).

Nanomedicine, compared to traditional drugs, demonstrates significant potential for treating MI due to its low toxicity and good biocompatibility. Among these, nanoceria stands out for its antioxidant properties in various pathological conditions. Its ability to switch oxidation states between Ce^3+^ and Ce^4+^ makes it a notable antioxidant with a high capacity for oxygen capture (Naganuma and Traversa, 2012; Khan et al., 2017; Yadav et al., 2019). The coexistence of dual oxidation states (Ce^3+^ and Ce^4+^) renders nanoceria redox-active, enabling the removal of both superoxide and hydrogen peroxide depending on the environment. To scavenge ROS, nanoceria mimics both superoxide dismutase (SOD) and catalase (CAT), with the former catalyzing the dismutation of superoxide (O_2_^−^) to hydrogen peroxide (H_2_O_2_) and molecular oxygen (O_2_), and the later decomposing hydrogen peroxide to oxygen and water (Baldim et al., 2018). In line with previous studies (Dewberry et al., 2022; Im et al., 2023), our research confirmed the presence of both Ce^3+^ and Ce^4+^ in ceria nanoparticles, which exhibited strong antioxidant activity. Furthermore, ceria nanoparticles not only act as direct antioxidants but also serve as ROS-based nanocarriers to mitigate oxidative stress.

Nrf2 is a critical transcription factor that maintains ROS homeostasis by regulating the transcription of multiple antioxidant genes (Li et al., 2019). Numerous studies, including ours, have demonstrated the essential role of Nrf2 in countering oxidative responses and cardiac remodeling post-MI. Nrf2 deficiency leads to higher mortality in mice post-MI, whereas activation of Nrf2 significantly improves cardiac function and reduces infarct size (Wu et al., 2022; Strom and Chen, 2017). However, Nrf2 is tremendously unstable and easily degraded (Sekhar et al., 2002). To address this, we constructed CeO_2_/Nrf2 nanocomposites using ceria nanoparticles to deliver Nrf2 plasmids, thereby enhancing Nrf2’s antioxidant function. The ceria nanoparticles effectively protect Nrf2 from degradation, deliver it to the heart, and increase its expression in both the cytoplasm and nucleus. Compared to ceria nanoparticles alone, the nanocomposites exhibited stronger cardioprotective effects, as evidenced by improved cardiac function and reduced fibrosis. Additionally, the nanocomposites demonstrated antioxidant and anti-inflammatory properties both *in vitro* and *in vivo* by enhancing Nrf2 expression and activity. Similarly, Zhang J. et al. synthesized porous magnetic silica nanoparticles loaded with sulforaphane, which activated Nrf2 and provided protection against MI in a mouse model (Zhang et al., 2023), further supporting the therapeutic potential of Nrf2-loaded nanoparticles for myocardial infarction.

We observed that, in addition to accumulating in the heart, CeO_2_/Nrf2 nanocomposites also distributed to other organs, though they did not exhibit toxicity to these organs. However, further efforts are needed to enhance the specificity of CeO_2_/Nrf2 nanocomposites to the heart post-MI. Additionally, innate immune cells are rapidly recruited to the infarct area at the onset of MI (Yap et al., 2023). Consequently, CeO_2_/Nrf2 nanocomposites can be promptly uptaken by macrophages and delivered to the infarct zone to prevent cardiomyocyte apoptosis. Nonetheless, it remains uncertain whether CeO_2_/Nrf2 nanocomposites would be equally effective in other cardiovascular diseases that lack an acute inflammatory response.

## 5 Conclusion

We successfully constructed CeO_2_/Nrf2 nanocomposites which could wrap Nrf2 plasmids and be engulfed by macrophage after tail vein injection and effectively deliver Nrf2 plasmids to the infarct zone. Consequently, Nrf2 was overexpressed in the heart post-MI, alleviating oxidative stress and inflammatory responses both *in vitro* and *in vivo*. Compared to CeO_2_ nanoparticles, CeO_2_/Nrf2 nanocomposites demonstrated superior effects in improving cardiac function, reducing cardiomyocyte apoptosis, and mitigating ROS and inflammation post-MI. Our findings suggest that CeO_2_/Nrf2 nanocomposites hold significant potential for the treatment of myocardial infarction.

## Data Availability

The original contributions presented in the study are included in the article/Supplementary Material, further inquiries can be directed to the corresponding authors.
