# Supplementary material for: Assembly of ceria-Nrf2 nanoparticles as macrophage-targeting ROS scavengers protects against myocardial infarction
Source: Front Pharmacol. 2025 Jan 10;15:1503757. doi: 10.3389/fphar.2024.1503757 (PMC11757866; doi:10.3389/fphar.2024.1503757)

Figure 2a. Example of original western blot for three repeats

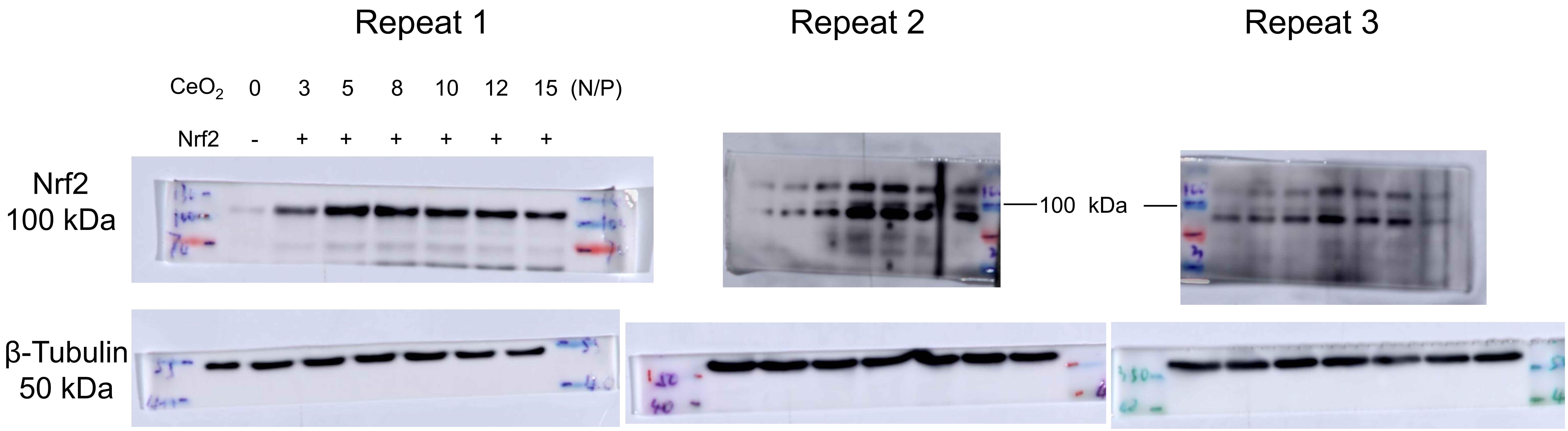

Figure 3c. Example of original western blot for three repeats

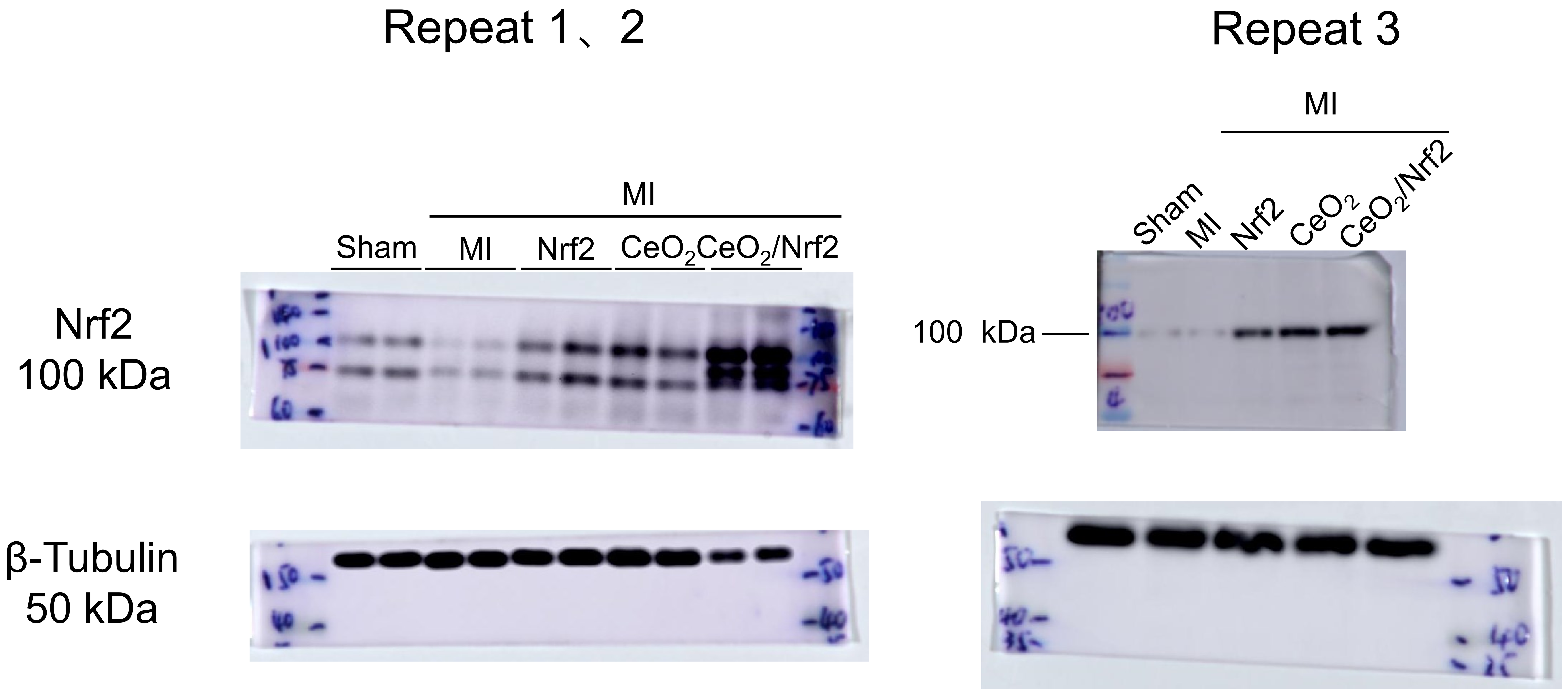

Figure 4d. Example of original western blot for three repeats

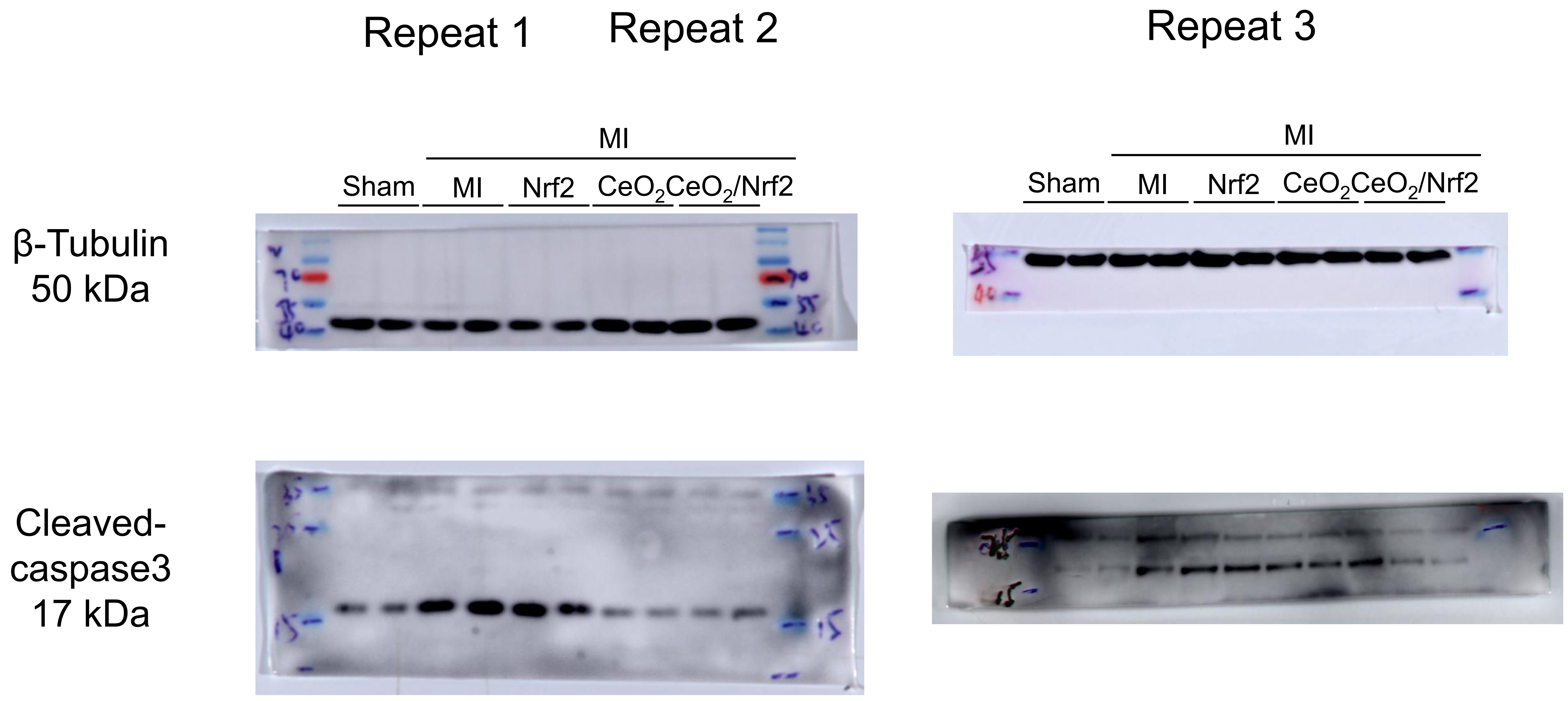

Figure 6a. Example of original western blot for three repeats

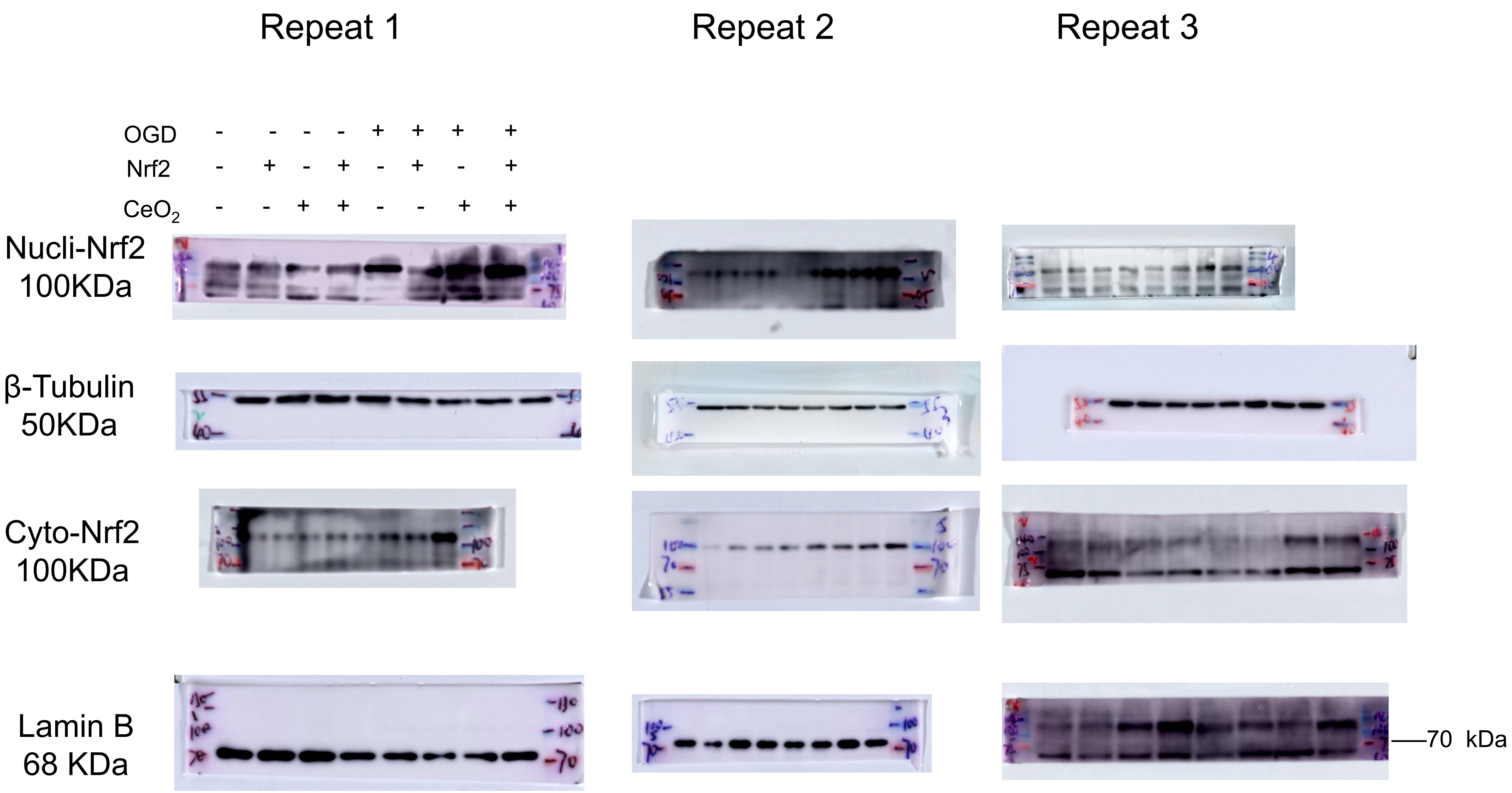

Figure 6d. Example of original western blot for three repeats

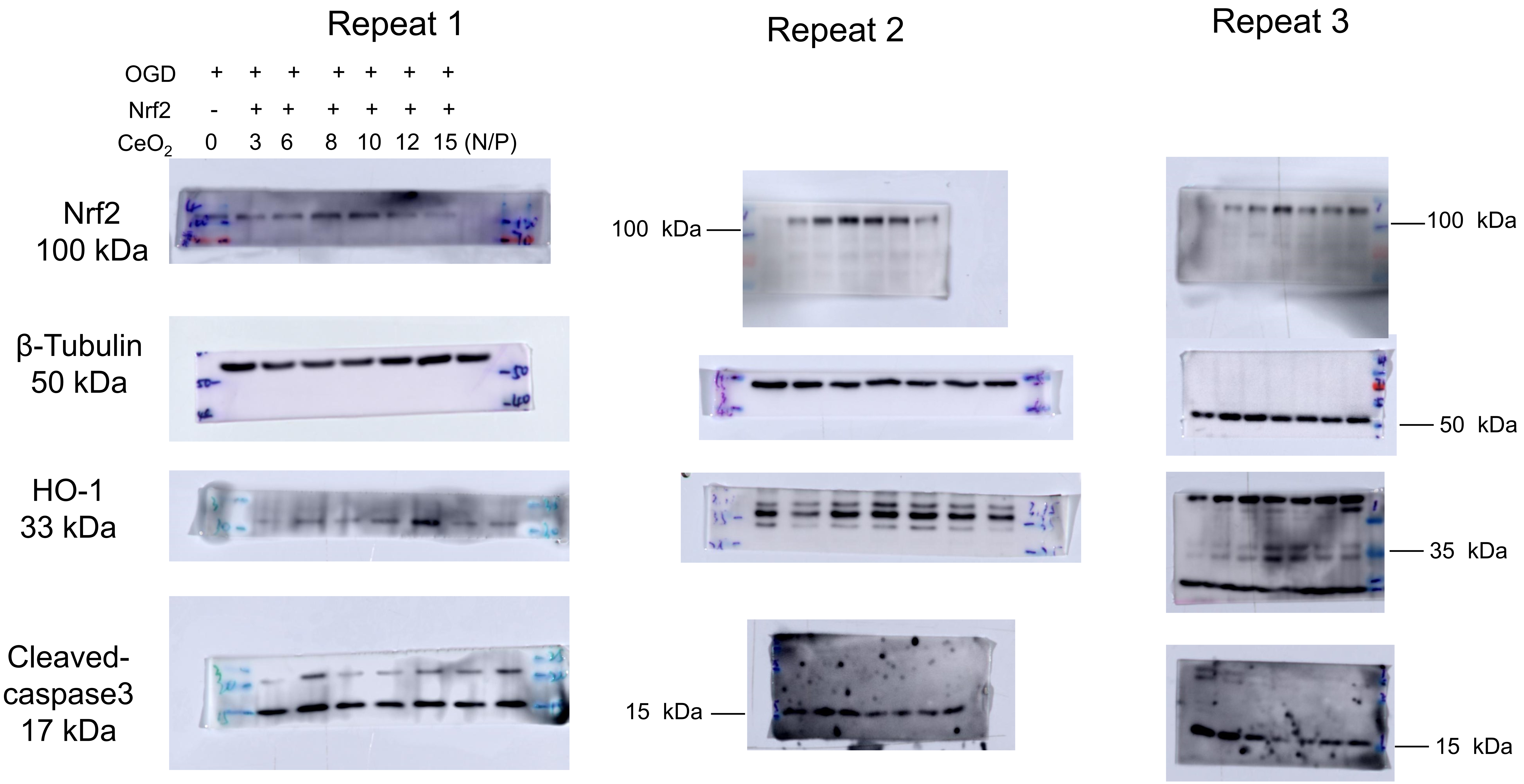

Figure S2a . Example of original western blot for three repeats

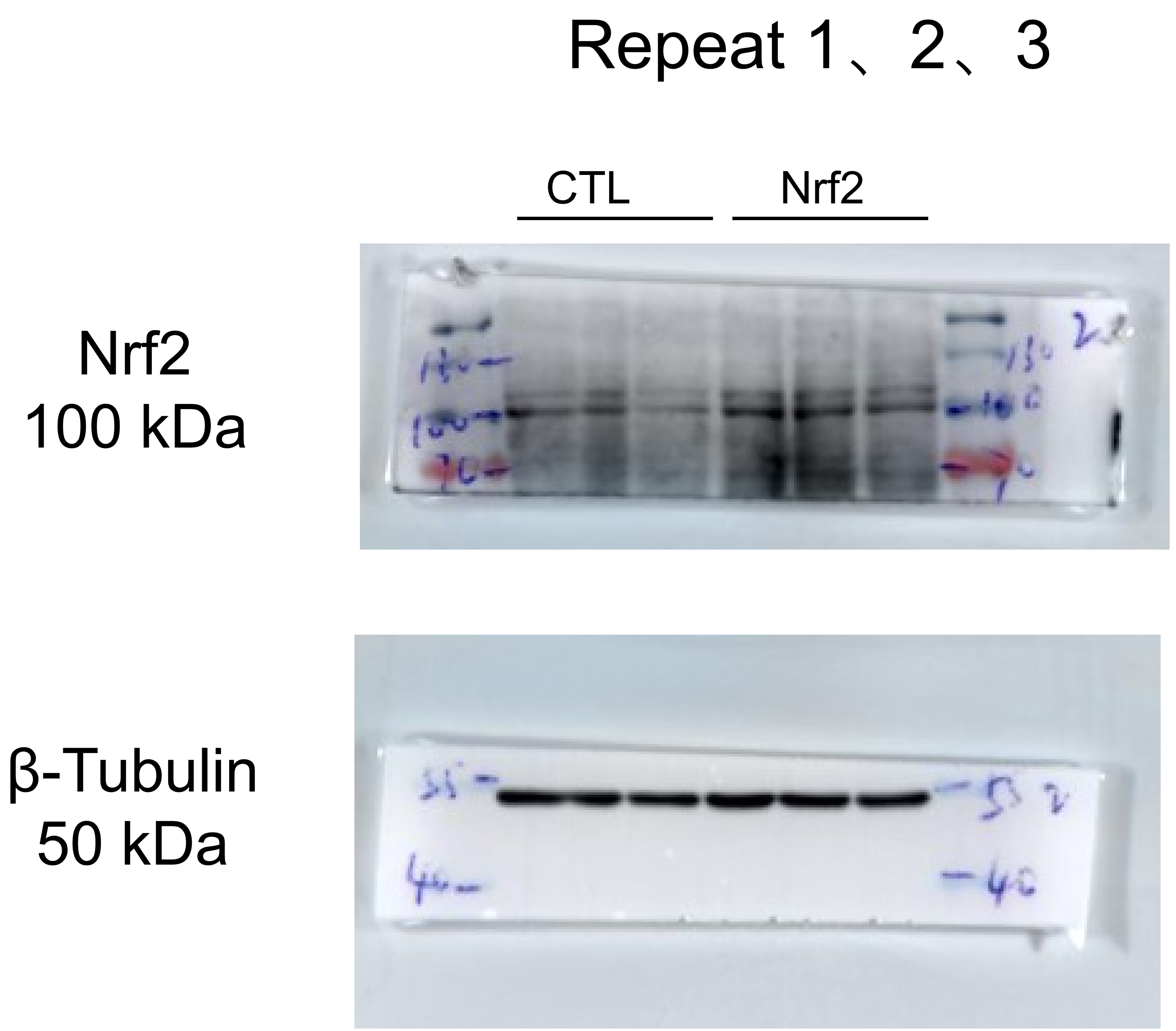

Supplement: Supplementary file 1 [file DataSheet2.pdf]
