# Supplementary material for: Assembly of ceria-Nrf2 nanoparticles as macrophage-targeting ROS scavengers protects against myocardial infarction
Source: Front Pharmacol. 2025 Jan 10;15:1503757. doi: 10.3389/fphar.2024.1503757 (PMC11757866; doi:10.3389/fphar.2024.1503757)
Supplement: Supplementary file 2 [file DataSheet1.pdf]

Supplementary Material

Assembly of Ceria-Nrf2 Nanoparticles as Macrophage-Targeting ROS Scavengers Protects against Myocardial Infarction

1. Materials

All primers were designed and synthesized by Sangon Biotech (Shanghai). The primer sequences were listed in table S1.

Table S1 The sequence of primers for qPCR

Gene name	**Forward (5’ - 3’)**	**Reverse (5’ - 3’)**
Gapdh	AGGTCGGTGTGAACGGATTTG	GGGGTCGTTGATGGCAACA
r-Nfe2l2	CACTCTGTGGAGTCTTCCATTT	GAATGTGTTGGCTGTGCTTTAG
HO-1	GTACACATCCAAGCCGAGAA	TGGTACAAGGAAGCCATCAC
NQO1	GAGAAGAGCCCTGATTGTACTG	ACCTCCCATCCTCTCTTCTT
TNF-α	CTGAGTTCTGCAAAGGGAGAG	CCTCAGGGAAGAATCTGGAAAG
IL-6	GAAGTTAGAGTCACAGAAGGAGTG (Sense)	GTTTGCCGAGTAGACCTCATAG (AntiSense)
TGF-β1	ACCTGTGCCTGCCATTAC	GTCCCTTACTTCCTGGCTTTAC

2. Results

H9C2 cardiac myoblasts were incubated with Ceria-Nrf2 nanoparticles at different N/P ratio. Cell viability of H9C2 cardiac myoblasts was assayed by using CKK-8 kit. As shown in Fig S1A, there is no obvious cytotoxicity of CeO2 nanoparticle in H9C2 cardiac myoblasts.

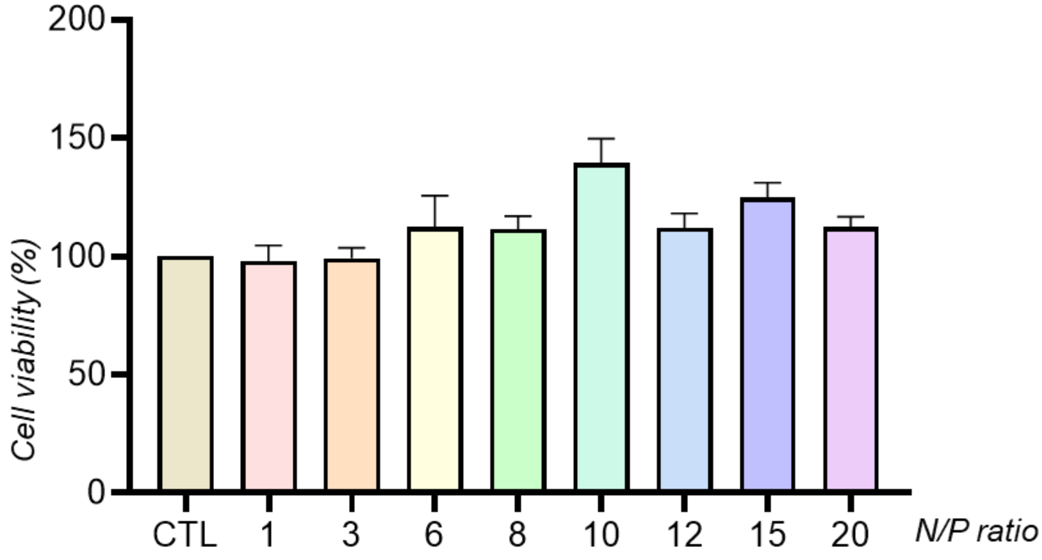

Fig. S1 The cell viability of H9C2 cardiac myoblasts.

H9C2 cardiac myoblasts were transfected with Nrf2 plasmids and the protein expression were measured and quantified by Western blot (Fig S2A). The CeO_2/Nrf2 nanocomposites or the CeO_2 nanomaterials was administrated to mice via tail vein injection within 0.5 - 1 h post MI. And the liver, spleen, kidney and lung were isolated. H&E staining results showed that CeO_2/Nrf2 nanocomposites did not alter the histological features of these organs (Fig S2B).

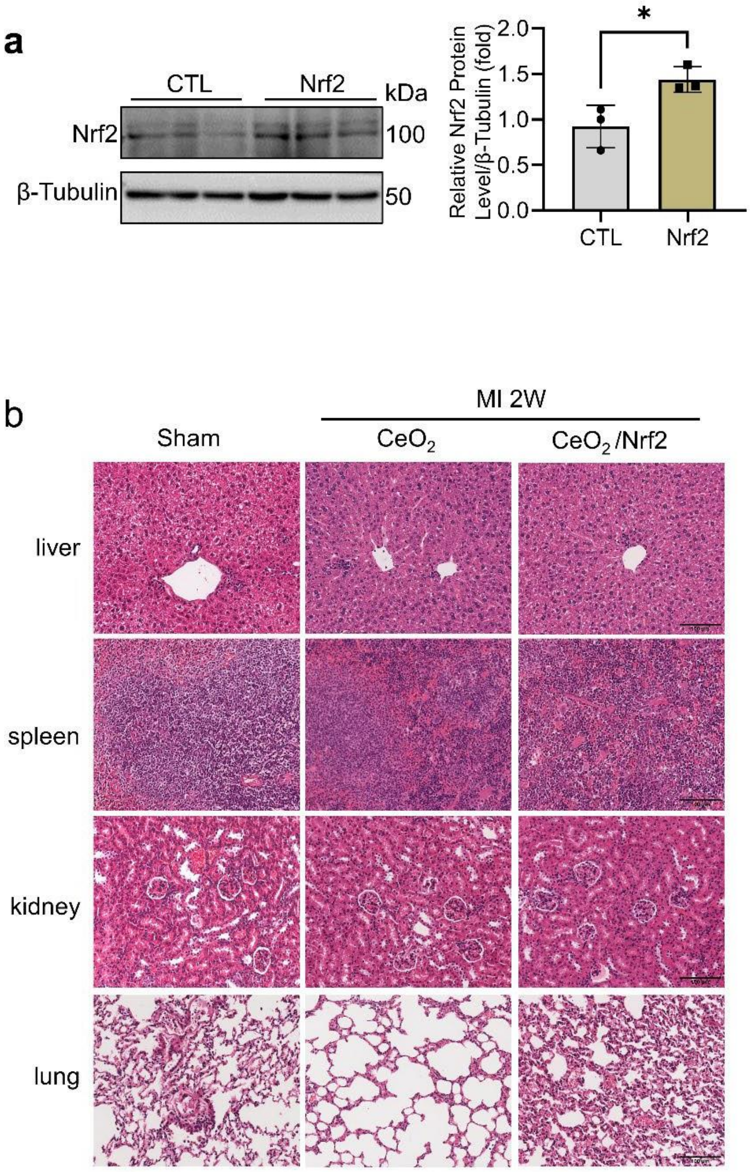

Fig. S2 (A) The protein expression of Nrf2 in H9C2 cardiac myoblasts. **(B)** Representative H&E staining images of liver, spleen, kidney and lung. Scale bar: 100 μm.

The Nrf2 plasmid, CeO_2 nanomaterials and CeO_2/Nrf2 nanocomposites were prepared, and administrated into MI mice by tail intravenous injection respectively within 0.5 to 1 hour post MI surgery. The administrations were repeated on day 1, day 3, day 5 and

day 7. We measured the cardiac function by transthoracic echocardiography at day 3 and day 14 post MI respectively.

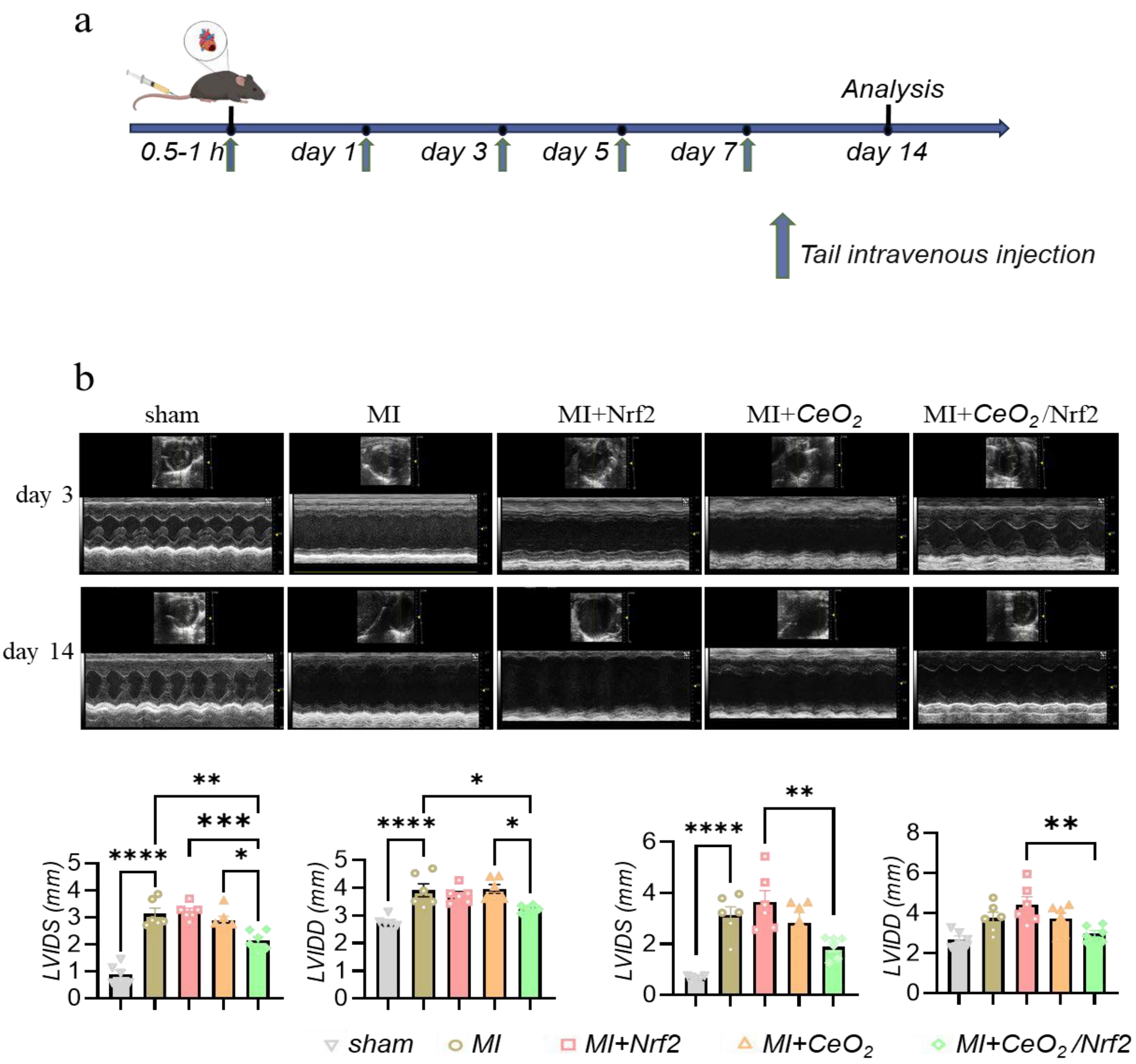

Fig. S3 (A) The scheme of this experiment. **(B)** Representative images of echocardiography and the quantifications of LVIDS, LVIDD, LVIDS and LVIDD. n=6. Data are presented as Mean ± SEM. One-way ANOVA test was used to detect significance.

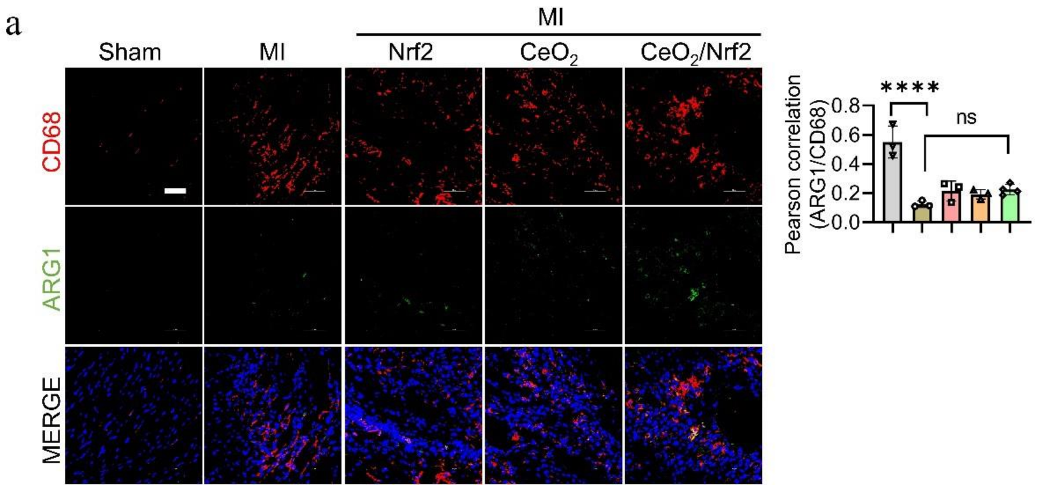

Fig. S4 (A) Representative image and analysis of Immunofluorescence staining CD68 expression (red) and M2 macrophage marker ARG1 (green) in the cardiac specimens of mice, scale bar, 50 μm, n = 3. Data are presented as Mean ± SEM. One-way ANOVA test was used to detect significance.

Table S2 Echocardiographic parameters

	sham (n=6)	MI (n=6)	MI+Nrf2 (n=6)	MI+CeO_2 (n=6)	MI+CeO_2/Nrf2 (n=6)
day 3					
HR (bpm)	548.8674±95.8997	559.6428±49.7668	542.0822±32.7348	571.9873±95.8997	541.6647±51.343
LVIDD (mm)	2.7282±0.2547	3.9109±0.5849****	3.747±0.2994	3.9601±0.2547	3.2399±0.1252$^{\#\&}$
LVIDS (mm)	0.8915±0.3568	3.1294±0.4996****	3.2096±0.2892	2.869±0.3568	2.144±0.3677$^{\#\#\$\$\$\&}$
LVAWD (mm)	0.9922±0.211	0.67±0.1714	0.5952±0.1458	0.6479±0.211	0.8446±0.3099
LVAWS (mm)	1.7088±0.4009	0.8307±0.1388****	0.6089±0.1323	0.7565±0.4009	1.0146±0.2833
LVPWD (mm)	1.0061±0.3315	0.6896±0.106	0.7948±0.1513	0.7056±0.3315	0.8427±0.0844
LVPWS (mm)	1.8298±0.2023	1.0276±0.3436***	1.0462±0.2825	1.1526±0.2023	1.281±0.2602
LVEF (%)	95.3481±3.6405	33.3035±5.2816****	33.0697±4.5252	42.4655±3.6405	66.5215±9.6128$^{\#\#\#\#\$\$\$\$\&\&\&}$
LVFS (%)	71.1479±8.4574	19.2365±4.8292****	19.9961±6.246	25.1227±8.4574	36.3487±7.5853$^{\#\#\#\$\$\&}$
day 14					
HR (bpm)	505.1599±81.9108	529.9012±44.9563	510.5927±93.5526	591.0681±23.6992	556.6712±39.5761
LVIDD (mm)	2.701±0.3913	3.7516±0.7134	4.4266±0.9644	3.7097±0.7159	2.9739±0.3938$^{\$\$}$
LVIDS (mm)	0.658±0.121	3.1378±0.783****	3.6347±1.116	2.8326±0.95	1.8823±0.4318$^{\$\$}$
LVAWD (mm)	0.858±0.2049	0.5726±0.1971*	0.5699±0.1621	0.6861±0.1212	0.7895±0.1267
LVAWS (mm)	1.5692±0.2016	1.2321±0.6616	0.5657±0.1439	0.8824±0.1936	1.412±0.1736$^{\$\$}$
LVPWD (mm)	0.8216±0.2293	0.5148±0.0768	0.7381±0.3119	0.7001±0.2476	0.6081±0.161
LVPWS (mm)	1.7965±0.138	0.7998±0.2505****	1.0326±0.4111	0.9673±0.2948	1.2533±0.2763
LVEF (%)	98.1314±0.8474	33.7979±12.770****	39.024±13.6992	45.3384±13.1739	67.8877±9.7108$^{\#\#\#\$\$\&}$
LVFS (%)	78.8726±3.3237	16.0063±6.9632****	19.097±7.3381	17.4663±6.6914	38.9358±7.0357$^{\#\#\#\#\$\$\$\&\&\&\&}$

Data are presented as Mean ± SEM. One-way ANOVA test was used to detect significance. $^{*}P < 0.05$, $^{**}P < 0.01$, $^{***}P < 0.001$, $^{****}p < 0.0001$ vs. sham group; $^{\#}P < 0.05$, $^{\#\#}P < 0.01$, $^{\#\#\#}P < 0.001$, $^{\#\#\#\#}P < 0.001$ vs. MI group, $^{\$}P < 0.05$, $^{\$\$}P < 0.01$, $^{\$\$\$}P < 0.001$, $^{\$\$\$\$}P < 0.0001$ vs. Nrf2 group, $^{\&}P < 0.05$, $^{\&\&}P < 0.01$, $^{\&\&\&}P < 0.001$, $^{\&\&\&}P < 0.0001$ vs. CeO_2 group, n = 6. HR, heart rate; LVIDD, left ventricular internal diastolic diameter; LVIDS, left ventricular internal systolic diameter; LVAWD, left ventricular end-diastolic anterior wall; LVAWS, left ventricular end-systolic anterior wall; LVPWD,

left ventricular end-diastolic posterior wall; LVPWS, left ventricular end-systolic posterior wall; LVEF, left ventricular ejection fraction; LVFS, left ventricular fractional shortening.
